# Looking for the Self: Phenomenology, Neurophysiology and Philosophical Significance of Drug-induced Ego Dissolution

**DOI:** 10.3389/fnhum.2017.00245

**Published:** 2017-05-23

**Authors:** Raphaël Millière

**Affiliations:** Faculty of Philosophy, University of OxfordOxford, United Kingdom

**Keywords:** self-awareness, minimal self, phenomenology, psychedelics, dissociatives, depersonalization, psychosis

## Abstract

There is converging evidence that high doses of hallucinogenic drugs can produce significant alterations of self-experience, described as the dissolution of the sense of self and the loss of boundaries between self and world. This article discusses the relevance of this phenomenon, known as “drug-induced ego dissolution (DIED)”, for cognitive neuroscience, psychology and philosophy of mind. Data from self-report questionnaires suggest that three neuropharmacological classes of drugs can induce ego dissolution: classical psychedelics, dissociative anesthetics and agonists of the kappa opioid receptor (KOR). While these substances act on different neurotransmitter receptors, they all produce strong subjective effects that can be compared to the symptoms of acute psychosis, including ego dissolution. It has been suggested that neuroimaging of DIED can indirectly shed light on the neural correlates of the self. While this line of inquiry is promising, its results must be interpreted with caution. First, neural correlates of ego dissolution might reveal the necessary neurophysiological conditions for the maintenance of the sense of self, but it is more doubtful that this method can reveal its minimally sufficient conditions. Second, it is necessary to define the relevant notion of self at play in the phenomenon of DIED. This article suggests that DIED consists in the disruption of subpersonal processes underlying the “minimal” or “embodied” self, i.e., the basic experience of being a self rooted in multimodal integration of self-related stimuli. This hypothesis is consistent with Bayesian models of phenomenal selfhood, according to which the subjective structure of conscious experience ultimately results from the optimization of predictions in perception and action. Finally, it is argued that DIED is also of particular interest for philosophy of mind. On the one hand, it challenges theories according to which consciousness always involves self-awareness. On the other hand, it suggests that ordinary conscious experience might involve a minimal kind of self-awareness rooted in multisensory processing, which is what appears to fade away during DIED.

## Introduction

Hallucinogenic drugs are known to produce profound changes in consciousness. While their immediate effects on perception and mood are well-documented (Halberstadt, 2015), some of their complex effects at higher doses have received less attention until more recently. In particular, a number of hallucinogenic compounds can induce thorough disturbances of self-consciousness, described as a dramatic breakdown of one’s sense of self, a phenomenon commonly referred to as “ego dissolution” (Lebedev et al., 2015). Similar disturbances of self-consciousness are also reported in several psychiatric disorders, specifically in acute psychosis (Bowers and Freedman, 1966; Gouzoulis-Mayfrank et al., 1998; Sass et al., 2013), as well as mystical-type experiences (Baumeister and Exline, 2002; Hood, 2002) and deep meditative states (Dor-Ziderman et al., 2013). However, in these endogenous cases, it is difficult to study the mechanisms of ego dissolution, due to its unpredictability and complex etiology. By contrast, the study of Drug-Induced Ego Dissolution (DIED) in healthy individuals offers a way to track down underlying mechanisms of ego dissolution, which in turn could help us gain a better understanding of the neurobiological basis of the sense of self.

This article presents an interdisciplinary perspective on DIED and its relevance in studying the sense of self, which is a multifaceted and controversial notion. First, the phenomenology of DIED will be described, based on an analysis of available subjective reports and quantitative data from questionnaires. It will be argued that DIED is a valid construct resulting from the use of psychoactive substances with different chemical structures. Second, this article will review current knowledge about the neurophysiology and neuropharmacology of DIED, which, albeit limited, offers promising insights into its neural underpinning. Third, this article will discuss the relevance of DIED for cognitive neuroscience. In particular, it will address methodological issues within studies investigating neural correlates of DIED, and the extent to which neuroimaging can shed light on the neurobiological basis of the sense of self. In addition, this article will examine the hypothesis that DIED consists in a disruption of the “embodied” or “minimal” self (the meaning of which will be discussed later) in light of phenomenological evidence, conceptual analysis and recent neurocomputational models of selfhood. Finally, it will be suggested that the study of DIED can also contribute to contemporary philosophical debates on self-awareness.

## The Phenomenology of DIED

The profound effects of hallucinogenic compounds on the sense of self have been well known for a long time, especially with regard to psychedelics. Some early experimental studies with mescaline (a psychedelic substance from the peyote cactus *Lophophora williamsii*) used it to model schizophrenic “self-disturbances”, including anomalous body experiences and the feeling of merging with one’s surroundings (Mayer-Gross and Stein, 1926; Beringer, 1927; see Mishara et al., 2016), while others concluded that it could replicate the symptoms of depersonalization disorder, characterized by the experience of feeling unreal and detached from one’s mental processes and body (Guttmann and Maclay, 1937). Aldous Huxley himself described his personal experimentations with mescaline as leading to a “final stage of egolessness”, associated with a feeling of unity with everything (Huxley, 1954). In the 1950s and 1960s, a host of experimental studies with the newly synthetized lysergic acid diethylamide (LSD) reached similar conclusions regarding the drug’s ability to produce depersonalization-like experiences. Reported effects of LSD in these studies include the blurring or complete disappearance of boundaries between one’s body and its environment, feelings of numbness, “feelings of non-existence”, fear of “impending dissolution”, feelings of disembodiment and feelings of unity with the universe (Anderson and Rawnsley, 1954; Savage, 1955; Bercel et al., 1956; Von Mering et al., 1957; Klee, 1963; Sedman and Kenna, 1964; Pahnke and Richards, 1966). Around the same time, similar effects were observed with the administration of psilocybin, the psychoactive component of so-called “magic mushrooms” found mainly in the *Psilocybe* genus (Rümmele and Gnirss, 1961; Pahnke, 1966). The expression “ego dissolution” seems to have emerged in the 50s to describe these effects as observed with LSD and mescaline (Anderson and Rawnsley, 1954; Denber, 1958; Lewis and Sloane, 1958; Cohen, 1960). Alternative expressions came into use in the late 50s and early 60s, such as “ego disintegration” (Eisner and Cohen, 1958; Kast and Collins, 1964), “ego loss” (Alnæs, 1964; Leary et al., 1964; Pahnke et al., 1969) and “ego death” (Alnæs, 1964; McGlothlin, 1964; Pahnke, 1969; Grof, 1970)^1^. Trying to relate DIED to better-known conditions, many of the studies published during this period compared the reported effects to the symptoms of depersonalization disorder or to putatively nonpathological mystical states. Several authors also described these effects as “self-disturbances” (*Ichstörungen*), a term introduced by Hans Gruhle (a colleague of Karl Jaspers in Heidelberg) to characterize passivity experiences in schizophrenia (Gruhle, 1915; see Mishara et al., 2016). The notion of “self-disturbance” was later systematized by Kurt Schneider, who included it among first-rank symptoms of schizophrenia, as “alterations of the sense of “I”, “me” or “mine” [*Meinhaftigkeit*]” linked to passivity symptoms (Schneider, 1959; see Bürgy, 2011; Mishara et al., 2014).

Later studies have attempted to capture the subjective effects of hallucinogenic drugs quantitatively by using standardized questionnaires. The use of psychometrics better ensured the validity and consistency of phenomenological reports within and between participants. One of the most widely used self-report questionnaires to measure the effects of psychoactive compounds is Dittrich’s Abnormal Mental States (APZ) questionnaire (Dittrich, 1975, 1996, 1998) and its revised versions, OAV (Bodmer et al., 1994) and 5D-ASC (Dittrich et al., 2006, 2010). These questionnaires measure the characteristics of altered states of consciousness along three primary dimensions: *Oceanic Boundlessness* (OBN), *Dread of Ego Dissolution* (DED) and *Visionary Restructuralization* (VR). The first two dimensions are loosely associated with positively and negatively experienced ego dissolution, respectively. The OBN scale includes items measuring positively felt depersonalization, derealization and feelings of unity (e.g., “It seemed to me that my environment and I were one”), whilst the DED scale measures distressing experiences of depersonalization, thought disorder, and loss of thought and body control (e.g., “I observed myself as though I were a stranger”). A large number of placebo-controlled studies have reported that healthy participants give on average statistically higher scores to items on the OBN and DED scales after the administration of various psychoactive drugs. Such drugs include: psilocybin (Hasler et al., 2004; Griffiths et al., 2006, 2011; Cummins and Lyke, 2013), *N,N*-Dimethyltryptamine (DMT, also found in the shamanic brew *ayahuasca*; Riba et al., 2002; Gouzoulis-Mayfrank et al., 2005; Alonso et al., 2015), ketamine (Vollenweider et al., 1997a; Northoff et al., 2005) and salvinorin-A (found in *Salvia divinorum*; González et al., 2006; Maqueda et al., 2015). A meta-analysis pooling raw data from eight experimental studies, conducted between 1999 and 2008, found that psilocybin significantly increased scores of all 5D-ASC scales in a dose-dependent manner, with higher doses (215 μg/kg and above) being associated with high scores of items in the OBN scale, including those related to “positively experienced depersonalization” (Studerus et al., 2011). Although a number of participants did not report significant alterations of self-experience, particularly when receiving lower doses of psilocybin, 22% of participants in the higher dose condition (315 μg/kg) scored above cut-off for experiencing strong OBN. A recent study used a 23-item rating scale questionnaire (based on previous empirical work by Carhart-Harris et al. (2012)) to assess the effects of 2 mg psilocybin. The authors found that after drug administration participants scored highly on the item “I experienced a disintegration of my self or ego” (Muthukumaraswamy et al., 2013). Another self-report questionnaire that used the Hallucinogen Rating Scale (HRS) yielded similar results regarding “subjective alterations [of] self-awareness” upon administration of psychoactive substances, such as DMT (Strassman et al., 1994) and salvinorin-A (Addy, 2011). In addition, several studies have used the Ego Pathology Inventory (EPI), a questionnaire initially designed to measure “self-disturbances” in schizophrenia (Scharfetter, 1981), to assess the effects of psychoactive compounds on self-consciousness. Vollenweider and colleagues have repeatedly found that ketamine and psilocybin administration significantly increased global EPI scores, including individual scores for the “ego demarcation” scale—measuring the blurring of boundaries between self and world—, as well as the “ego consistency” scale—measuring the dissolution of the sense of being a single coherent self (Vollenweider et al., 1997a,b,c; Vollenweider and Geyer, 2001).

One noteworthy limitation of most recent studies directly investigating the phenomenon of ego dissolution, however, is their reliance on a single questionnaire item to measure it, such as “I experienced a disintegration of my self or ego” (Muthukumaraswamy et al., 2013; Carhart-Harris et al., 2014, 2016; Lebedev et al., 2016; Tagliazucchi et al., 2016). Not only are single-item measurements less reliable than scales with multiple items, by virtue of their limited ability to capture the complex phenomenology of DIED, but they are also more ambiguous, as participants might have widely different understandings of the notions of “self” or “ego”. In addition, items of the 5D-ASC questionnaire itself appear to measure DIED only indirectly, through measuring experiences of unity (“Everything seemed to unify into an oneness”) and disembodiment (“It seemed to me as though I did not have a body anymore”) (Studerus et al., 2010). In a recent double-blind, placebo-controlled study focusing specifically on DIED and its neural correlates, 15 participants completed a custom questionnaire made of 22 Visual Analogue Scale (VAS) items after receiving an injection of 2 mg psilocybin (Lebedev et al., 2015). Rather than using a single-item measurement of DIED, the questionnaire data was reduced using Principal Component Analysis (PCA), a statistical procedure used to identify the principal components of a multidimensional dataset accounting for as much of the variability in the data as possible, under the constraint that variables correlate with each other in each principal component. PCA yielded a first principal component which was mainly associated with VAS items describing ego dissolution, such as “I lost all sense of ego” (Spearman’s rank correlation coefficient *r* = 0.842), “I experienced a loss of separation from my environment” (*r* = 0.756) and “I felt like I was merging with my surroundings” (*r* = 0.717). The authors interpreted this convergence as evidence that DIED is a valid construct and corresponds to a meaningful aspect of the subjective experience of participants. Although this result is very promising, it should be noted that the first principal component in this study was also associated with items related to visual abnormalities, such as “I saw geometric patterns” (*r* = 0.725) and “My imagination was extremely vivid” (*r* = 0.841). Furthermore, the authors performed PCA on ratings from their own unvalidated questionnaire.

These limitations were recently addressed in a timely article introducing a new 8-item “Ego Dissolution Inventory” (EDI) specifically designed to measure DIED (Nour et al., 2016). In this study, 691 participants completed an online VAS questionnaire including eight items relating to ego dissolution, and eight items relating to “ego inflation” (construed as an “experience of increased self-assuredness”) for experiences with classical psychedelics, cocaine and alcohol, as well as items selected from the Mystical Experience Questionnaire (MEQ) relating to experiences of unity. The goal was to assess the construct validity of the 8-item EDI, by demonstrating both its discriminant validity against the 8 ego inflation items, and its convergent validity with the MEQ items. The authors used exploratory factor analysis (EFA) to identify latent constructs underlying questionnaire items in a hypothesis-free manner. The analysis supported a model with two main orthogonal factors, respectively accounting for 36.6% and 29.5% of the variance in the sample, and respectively associated with the eight “ego dissolution” and the eight “ego inflation” items (each item loading strongly onto one of the two factors), thus demonstrating the discriminant validity of the 8-item EDI. Moreover, the score for MEQ-based measure of unitive experience was found to be strongly correlated with the 8-item EDI, demonstrating its convergent validity. This suggests that the proposed EDI is internally valid, has a single-factor psychometric structure and measures a valid construct. The items which loaded the most strongly onto the “ego dissolution” factor (*r* > 0.800) were “I experienced a disintegration of my “self” or ego” (*r* = 0.897), “I lost all sense of ego” (*r* = 0.885), “I experienced a dissolution of my “self” or ego” (*r* = 0.883), “All notion of self and identity dissolved away” (*r* = 0.845) and “I felt at one with the universe” (*r* = 0.830). In line with previous studies using the ASC questionnaire, the EDI score was found to be strongly associated in a dose-dependent manner with experiences induced by classical psychedelics, as opposed to those induced by cocaine and alcohol. Two potential limitations of the EDI should be noted. First, given that several items are extremely similar, it is not very surprising that they should load onto the same factor. Second, like unvalidated questionnaires, the EDI might be limited by the vagueness of its two key terms, namely “ego” and “self”. Thus, although it measures a valid construct, it does not seem able to shed light on the nature of DIED beyond its standard account as a loss/disintegration/dissolution of the self/ego. Nonetheless, this study represents an important step in the demonstration that DIED is a genuine effect of at least some psychotropic substances, and introduces a reliable way to measure it.

An alternative way to get some insight into the phenomenon of DIED is to analyze anecdotal self-reports from drug users, such as those found on the website Erowid.org^2^. Erowid.org is a curated database hosting more than 20,000 “trip reports” with various psychoactive substances. A quantitative analysis of randomly selected trip reports regarding Salvia yielded significantly high score for the category “ego dissolution” (Stiefel et al., 2014; it should be noted that the authors used their own measure of ego dissolution with items borrowed from several existing inventories as well as new items). For the present article, a new quantitative analysis of thousands of reports from Erowid.org was performed, using latent Dirichlet allocation (Blei et al., 2003) to extract semantic patterns from the corpus. The resulting topic model suggested that around 7% of trip reports concerning *Psilocybe* mushrooms, LSD, Salvia, DMT, 5-MeO-DMT, ayahuasca and ketamine involve the description of an ego dissolution experience. Frequently described aspects of DIED, within this subset of reports, include the loss of self-awareness (“I felt as if “I” did no longer exist”), the feeling of dying (“I felt like I was dying”), the loss of boundaries between self and world (“I felt myself mold into the world around me”), the loss of sense of bodily ownership (“I felt disconnected from my body”) and the failure to recognize oneself in a mirror (“I looked at myself in the mirror, only to see a person I could not even recognize”). Thus, the computer-assisted analysis of narrative reports is broadly consistent with questionnaire-driven studies in suggesting that DIED involves a dissolution of self-awareness and a feeling of unity with one’s surroundings (or indeed the whole universe). While reports obtained from Erowid.org are somewhat noisy and potentially biased, it should be noted that the unprecedented sample size (thousands of reports), as well as the use of a sophisticated machine learning algorithm extracting topics in a bottom-up (i.e., unsupervised) fashion, increase the significance of the results (see also Coyle et al., 2012 on another use of machine learning with Erowid.org).

Therefore, early anecdotal reports, data from validated questionnaires and the above quantitative analysis of narrative reports, all converge to indicate that a range of psychoactive substances, especially above a certain drug-dependent dosage threshold, significantly alter the sense of self in similar ways. However, even if these effects exist, a question lingers about whether or not DIED is a unified phenomenon, rather than a cluster of heterogeneous subjective effects with potentially distinct underlying mechanisms. Two main reasons may warrant such a query: first, the effects associated with DIED are reported with a multiplicity of drugs which have different psychopharmacological profiles; second, the primary dimensions of the APZ questionnaire and its variants (such as 5D-ASC) seem to indicate that the OBN and DED item clusters, both associated with ego dissolution, measure two distinct and potentially incompatible subsets of effects. The first worry will be discussed in the next section of this article. As for the second, it stems from the fact that OBN and DED are respectively designed to measure positive and negative aspects of the experience. This could simply mean that DIED can be experienced in a positive or negative way, the main difference between the two scales lying in the emotional valence of the drug-induced state, not in the type of the subjective experience. Indeed, a number of external factors unrelated to the drug type and dosage, such as personality traits, previous experience, expectations, mood and environment have been shown to strongly predict the pleasant or unpleasant character of the experience (Metzner et al., 1965; Studerus et al., 2012). Due to its dramatic intensity, DIED can be a very frightening experience, as evidenced by the large number of individuals reporting a fear of dying. It is not surprising, then, that it is sometimes associated with severe anxiety, which can be greatly alleviated by the willingness to surrender to the experience (e.g., an individual writes in a typical Erowid.org report “I truly had to let go of myself, and that was profound”). Experienced users seem to have much more positive experiences with DIED on average, as they do not fear for their lives and are not trying to resist the effects of the drug. This could also explain why endogenously occurring ego dissolution, specifically in acute psychotic episodes, generally scores high on the DED dimension of the APZ questionnaire. In other words, when ego dissolution is expected, or at least relatable to drug intake, it is less likely to be associated with anxiety, and more likely to be a blissful experience. This is consistent with the significant positive correlation observed by Nour et al. (2016) between EDI score and long-term well being and life satisfaction (it should be noted than more than 50% of the participants had used classical psychedelic drugs on more than 10 occasions). Besides, even in endogenous cases, the emotional valence of the experience is also mediated by contextual factors, and accordingly psychotic ego dissolution is sometimes reported as a pleasant experience also (Gouzoulis-Mayfrank et al., 1998). This suggests that it would be wrong to put too much stress on the distinction between *OBN* and *DED*, while associating the first dimension with psychedelics and the second with psychotic episodes.

A recent psychometric evaluation of the APZ questionnaire, and its revised versions OAV and 5D-ASC, found that the three-dimensional organization of the items was not ideal to measure and compare altered states of consciousness accurately (Studerus et al., 2010). Instead of the three initial dimensions, a thorough statistical analysis yielded 11 clusters formed from 42 of the 66 original items, two of which seem related to ego dissolution: “Experience of unity” (with items such as “It seemed to me that my environment and I were one”) and “Disembodiment” (with items such as “I seemed to me as though I did not have a body anymore”). In this reorganized model, item clusters related to the affective dimension of the experience, such as “Spiritual experience” and “Anxiety”, were independent. A similar conclusion was reached by Lebedev et al. (2015) after reducing their custom questionnaire data with PCA. The authors found that ego dissolution, as measured by their first principal component, was “relatively independent from the emotional valence of the experience” (p. 12). Indeed, they reported that higher scores with the first principal component did not strongly correlate with the pleasant or anxious character of the experience—which correlated instead with the second principal component of PCA, orthogonal to the first. It should be noted, however, that the first principal component in this study was also loosely associated with two items related to anxiety, “I felt afraid” (*r* = 0.675) and “I feared loosing control of my mind” (*r* = 0.665). Nonetheless, there seems to be convergent evidence to support the idea that DIED can be experienced as a terrifying or blissful state, depending on the context and previous experience of individuals.

## The Neurophysiology of DIED

The term “hallucinogen” does not define a homogenous class of molecules, and is often liberally used to refer to pharmacologically active substances that alter consciousness in dramatic and somewhat unpredictable ways (Nichols, 2004). The subset of hallucinogens that have been shown to induce ego dissolution experiences is not homogenous either, and can be broken down into three main neuropharmacological categories: psychedelics, dissociative anesthetics and kappa opioid receptor (KOR) agonists.

### Classical Psychedelics

The most prominent neuropharmacological category is the class of “classical hallucinogens”, also referred to as (classical) “psychedelics” (Osmond, 1957). Psychedelics share the commonality that they act on the central nervous system (CNS) as agonists of the serotonin 2A (5-HT_2A_) receptor subtype (Glennon et al., 1984; Titeler et al., 1988; Sadzot et al., 1989). Although they come from multiple chemical families (tetracyclic ergolines such as LSD, indolealkylamines such as psilocybin and DMT, and phenylalkylamines such as mescaline, Halberstadt, 2015), they produce remarkably similar subjective effects, as evidenced by the difficulty of experienced users to discriminate between them in a blind setup (Wolbach et al., 1962). In particular, they produce strong visual effects, such as simple and complex hallucinations, which are less prominent in other classes of psychoactive substances (Gouzoulis-Mayfrank et al., 2005; Studerus et al., 2010). While phenylalkylamine hallucinogens are selective for 5-HT_2_ serotonin receptor subtypes, indolealkylamine hallucinogens bind non-selectively to all 5-HT receptors (Halberstadt, 2015). There is also converging evidence that 5-HT_2A_ receptor activation is specifically responsible for mediating the main subjective effects of all psychedelics (Glennon et al., 1984; Halberstadt, 2015). Indeed, Vollenweider et al. (1998) have shown that the effects of psilocybin are completely blocked by pre-treatment with ketanserin (a 5-HT_2A/2C_ antagonist) and risperidone (a mixed 5-HT_2A_/D_2_ antagonist), while pre-treatment with haloperidol (a dopamine D_2_ antagonist with weaker affinity for the 5HT_2A_ receptor) had no effect. Thus, psychedelics can be succinctly defined as serotoninergic hallucinogens, as opposed to other psychoactive substances, which primarily act on different neurotransmitter receptors (such as glutamate and opioid receptors).

The neurophysiological effects of serotoninergic hallucinogens are complex. 5-HT_2A_ receptors are expressed throughout the cortex (Pazos et al., 1987), and are most densely present in high-level association cortical areas associated with cognitive and perceptual processing (Carhart-Harris et al., 2012). In terms of the laminar structure of the cortex, 5-HT_2A_ receptors are most densely expressed on excitatory layer V pyramidal neurons (Weber and Andrade, 2010), and a recent MEG study suggests that serotoninergic hallucinogens might particularly activate these neurons (Muthukumaraswamy et al., 2013). Excitation of deep layer V pyramidal neurons is likely to be the cause of the desynchronization of cortical activity recorded by EEG and MEG in multiple studies after the administration of psilocybin, LSD and ayahuasca (Bente et al., 1958; Riba et al., 2002; Muthukumaraswamy et al., 2013). Decreases in cerebral blood flow and BOLD signal post-psilocybin were localized in high-association “hubs” of the cortex with a high concentration of 5-HT_2A_ receptors, such as the thalamus, the anterior and posterior cingulate cortices (ACC and PCC) and the medial prefrontal cortex (mPFC; Carhart-Harris et al., 2012; Muthukumaraswamy et al., 2013). These regions include key cortical nodes of the default mode network (DMN; Buckner et al., 2008), a large-scale intrinsic network which is highly active at rest but less active during goal-directed tasks (Raichle et al., 2001). It has recently been shown that oral ingestion of ayahuasca (containing DMT) also results in a reduction of DMN activity (Palhano-Fontes et al., 2015). The DMN is especially engaged in higher-order, metacognitive operations such as introspection and memory retrieval (Qin and Northoff, 2011), and many authors have suggested that the cortical midline structures of the brain, such as the mPFC, the PCC and the ACC, are associated with self-related processing in general (e.g., Gusnard et al., 2001; Northoff and Bermpohl, 2004; Ochsner et al., 2005). It is worth noting that DMN activity is also reduced during deep meditative states (Brefczynski-Lewis et al., 2007; Brewer et al., 2011), often associated with reports of ego dissolution (Dor-Ziderman et al., 2013).

Several additional findings by Carhart-Harris et al. (2013) have linked the neurobiological effects of psychedelics to the DMN. While the activation of the DMN is normally negatively correlated with the activation of the task-positive network (TPN), a network of regions that are consistently activated during goal-directed cognition, an increase in DMN-TPN functional connectivity was observed post-psilocybin. A decrease in DMN-TPN inverse coupling has also been observed during meditation, especially with techniques from Vedic and Chinese traditions belonging to the category of “non-dual awareness” meditation, which aims at the disappearance of the dichotomy between subject and object (Josipovic et al., 2012). Accordingly, it has been suggested that DMN-TPN orthogonality or “anti-correlation” might be necessary to maintain a clear distinction between what is internal/self-related and what is external/other-related at the personal level, which would be consistent with the occurrence of ego dissolution after the administration of psychedelics (Carhart-Harris et al., 2014). Functional connectivity analyses also revealed that psilocybin decreases coupling between the PCC and the mPFC, and increases coupling between the mPFC and task-positive areas such as the dorsolateral prefrontal cortex (dlPFC). Given the association of PCC activity with self-related processing, it has been suggested that PCC-mPFC decoupling might play a role in the etiology of DIED (Brewer et al., 2013). Carhart-Harris and colleagues also observed a highly significant positive correlation between decrease in alpha oscillatory activity in the PCC and ratings of the self-report item “I experienced a disintegration of my ‘self’ or ‘ego”’ post-psilocybin (Muthukumaraswamy et al., 2013; Carhart-Harris et al., 2014). This indicates that PCC alpha rhythms may play a role in the maintenance of the sense of self. Finally, functional connectivity analyses of the hippocampal region have revealed that psilocybin decreases functional coupling between the nodes of the DMN and the hippocampus and parahippocampal structures of the medial temporal lobes (MTLs; Carhart-Harris et al., 2014). Electrical stimulation of MTL regions is known to produce dream-like states associated with depersonalization-like experiences (Halgren et al., 1978). Moreover, neuroimaging has linked depersonalization disorder to the abnormal functioning of these regions (Lemche et al., 2016). This suggests that DMN-MTL decoupling might play a role in DIED.

According to the free-energy principle, the brain is a self-organizing system regulated by the minimization of its free energy (Friston, 2010). Interpreting the changes in brain connectivity post-psilocybin in light of this principle, Carhart-Harris and colleagues have suggested that serotoninergic hallucinogens produce a “disorganized” brain state characterized by a higher neural entropy (a dimensionless quantity measuring disorder) than normal waking consciousness (Carhart-Harris and Friston, 2010; Carhart-Harris et al., 2014). Accordingly, they hypothesized that the emergence of a coherent sense of self is underpinned by the self-organizing activity of the DMN, associated with a state of lower entropy, and that DMN-MTL decoupling plays an important role in the drug-induced phase transition to a “primary state” of consciousness in which DIED is more likely to occur. Given that the parahippocampal cortex (PHC) mediates the connectivity of the hippocampus with major network hubs, including the PCC (part of the DMN) and the dorsomedial prefrontal cortex (dMPFC), they specifically predicted that decoupling of the PHC from the neocortex would correlate with DIED. These hypotheses were tested in a recent study using a custom unvalidated questionnaire (including the item “I lost all sense of ego”) to investigate the neural correlates of DIED with 15 participants who received 2 mg psilocybin (Lebedev et al., 2015). Two main orthogonal factors were extracted from the item scores using PCA, and the first principal component was hypothesized to be associated to DIED (although it was also associated to items pertaining to perceptual abnormalities). Using the first principal component as a measurement of ego dissolution, the intensity of DIED was found to be strongly anticorrelated to the diversity of connections in the anterior part of the PHC (aPHC), which has specially been linked to dreamy states (Bartolomei et al., 2012); since the PHC mediates the connectivity of the hippocampus with the DMN (Ward et al., 2014), this result confirms the predicted association between DIED and MTL-DMN decoupling. On the other hand, the diversity of connections in the posteriomedial cortex (including the PCC) did not correlate with DIED, but rather with the negative emotional valence of the experience (hypothetically measured by the second principal component of PCA). Lower diversity of the left frontoparietal regions at baseline was found to indicate a higher likelihood of experiencing DIED. On a larger scale, DIED was found to be associated to the disconnection of MTL regions from the neocortex, as predicted. Surprisingly, the association between DIED and MTL-neocortex decoupling was more generalized than anticipated, since DIED did not correlate with disintegration of the DMN. However, it did correlate with disintegration of the salience network, which was unexpected. The salience network, which enables switching between the default mode and task-related states of brain connectivity, has independently been put forward as a potential candidate in the search for neural correlates of the “embodied self” (Craig, 2009; Seth, 2013), while the DMN might be more closely related to the “narrative self” given its role in introspection. Finally, DIED was also found to correlate with reduced interhemispheric connectivity, which was not expected either; this result appears to be consistent with a hypothesis regarding the role of interhemispheric communication in the maintenance of bodily awareness and one’s sense of agency (Uddin, 2011).

Several recent studies have also investigated the correlates of LSD-induced ego dissolution, using single-item measurements rather than the heterogeneous PCA-driven measurement previously used by Lebedev et al. (2015). Administration of LSD in healthy participants was found to be associated with an increase in global functional connectivity in high-level association cortices (which partially overlap with the DMN, the salience network and the frontoparietal attention network), as well as in the thalamus (Tagliazucchi et al., 2016). Interestingly, the experience of ego-dissolution and its intensity (as measured by the VAS questionnaire item “I experienced a disintegration of my ‘self’ or ‘ego”’) was found to correlate with this increase in global connectivity, specifically in the bilateral temporo-parietal junction (TPJ; angular gyrus) and bilateral insular cortex. Furthermore, the increase in global connectivity in high-level regions particularly involved sensory areas. The TPJ is an important area for multisensory integration and vestibular processing (Calvert et al., 2000; Bremmer et al., 2002), and its activity has been associated with changes in self-location and in the experienced direction of visuospatial perspective induced by full-body illusions with a head-mounted display (Ionta et al., 2011). Furthermore, it has been hypothesized that out-of-body experiences (OBE), in which individuals feel located outside of their own physical body (usually at an elevated position) stem from a failure to integrate multisensory information from one’s own body at the TPJ (Blanke and Arzy, 2005). A lesion analysis of OBE-patients also revealed that maximal lesion overlap at the TPJ included the angular gyrus (Ionta et al., 2011). A recent review suggests that the TPJ might contribute to the maintenance of a sense of self (Eddy, 2016). The insular cortex, in turn, has been associated with processing of interoceptive information (Craig, 2009), which might also play an important role in the experience of body ownership (Aspell et al., 2013; Suzuki et al., 2013) and the maintenance of a sense of self (Seth, 2013). Heautoscopic experiences, in which patients may feel located at several places at the same time, have been linked to lesions of the posterior insular cortex, and might be caused by the disintegration of visuo-somatosensory signals with interoceptive signals (Heydrich and Blanke, 2013). In light of these results it is tempting to speculate, as Tagliazucchi et al. (2016) do, that increased cross-talk between the angular gyrus, the insula and other brain networks plays a role in the experience of DIED. A separate analysis of the same fMRI data also found that DIED (as measured by the same single VAS item) was correlated with a disintegration of the DMN, i.e., a decrease in resting state functional connectivity within the network, supporting some previous findings with psilocybin (Carhart-Harris et al., 2014)—but not the results of the PCA-driven study of DIED (Lebedev et al., 2015). DIED was also found to be strongly correlated with a decrease in resting state functional connectivity between the parahippocampus and retrosplenial cortex. MEG revealed a significant relationship between DIED and decreased delta and alpha power, particularly in the PCC, also in line with previous research on psilocybin (Muthukumaraswamy et al., 2013). The authors thus suggest that DMN integrity, communication between the parahippocampus and the retrospenial cortex and regular oscillatory rhythms within the PCC may play a role in the maintenance of a sense of self. Finally, another recent study found that the increase in entropic brain activity produced by LSD predicted a long-term increase in the personality trait openness (Lebedev et al., 2016). Moreover, the predictive power of entropy was greatest for participants who had experienced ego-dissolution.

To summarize these findings, fMRI data with psilocybin suggested that ego dissolution is linked to MTL-cortical coupling, salience network integrity and interhemispheric connectivity. However, MTL-neocortex decoupling was not emphasized in recent studies on LSD-induced ego dissolution. Rather, these studies linked DIED to increased global cortical connectivity and increased entropy. There is nonetheless a fair share of overlap between the results of studies with psilocybin and LSD, in particular the correlation of DIED with decreased regular oscillatory rhythms in the PCC, and decreased communication between the parahippocampus and the neocortex (e.g., the retrosplenial cortex).

### Dissociative Anesthetics

Dissociative anesthetics form the second class of psychoactive substances known to induce ego dissolution experiences. The common characteristic of dissociative anesthetics such as ketamine, phencyclidine (PCP), dizocilpine (MK-801) and dextromethorphan (DXM) at subanesthetic doses is their primary action as antagonists of the* N-*methyl-D-aspartate (NMDA) glutamate receptor (Anis et al., 1983). The subanesthetic effects of dissociative anesthetics resemble some of the positive, negative and cognitive symptoms of schizophrenia (Lahti et al., 1995; De Simoni et al., 2013), and seem to include ego dissolution (Vollenweider et al., 1997b). These psychotomimetic characteristics have led a lot of researchers to use NMDA receptor antagonists to model the neurobiological mechanisms of psychosis, culminating with glutamatergic models of schizophrenia, according to which it is linked to NMDA receptor hypofunction (Javitt et al., 2012). Indeed, it is believed that the effects of dissociative anesthetics on consciousness and cognition are mediated by an increase in glutamate release resulting from NMDA receptor blockade, which leads to excessive glutamate activity at non-NMDA receptors (Krystal et al., 1994). The hypothesis that ketamine acts through increased glutamate release was supported by the finding that pre-treatment with lamotrigine, a drug reported to inhibit presynaptic glutamate release, significantly reduces the psychosis-like effects of ketamine (Anand et al., 2000). There is also increasing evidence that glutamatergic dysfunction might be involved in the pathophysiology of depersonalization disorder (Chambers et al., 1999; Sierra, 2008). For example, two open-label trials used lamotrigine as an add-on medication with antidepressants and found that 50%–70% of depersonalized patients reported improved symptoms (Sierra et al., 2001, 2006).

NMDA receptors are widely distributed in the cortex as well as the hippocampus and the amygdala. In rats, ketamine was shown to significantly increase glutamate release in the mPFC (Moghaddam et al., 1997). There is also converging evidence that NMDA receptor antagonists, just like serotoninergic hallucinogens, have a significant disruptive effect on PCC activity. An fMRI study on the long-term effects of ketamine in primates found that chronic ketamine administration reduced activity in the ventral tegmental area, the substantia nigra, the PCC and the visual cortex. Ketamine also increased activity in the striatum and entorhinal cortex (Yu et al., 2012). Previous studies had found that PCP, MK-801 and ketamine can induce severe metabolic reduction in the PCC (Olney et al., 1989; Sharp et al., 1991). More recently, a study found that during episodic memory retrieval ketamine significantly reduced the activation and the signal changes in the PCC, which correlated positively with psychosis-like symptoms (Northoff et al., 2005), in accordance with reported alterations of the glutamatergic system in the PCC of schizophrenic patients (Newell et al., 2005). Deakin et al. (2008) performed two experiments using resting-state fMRI to investigate directly the effects of ketamine on regional BOLD signal. In the first experiment, healthy participants received either intravenous ketamine or saline placebo. In the second experiment, participants were pretreated with lamotrigine before ketamine administration. This pre-treatment was done in order to identify the regional effects of ketamine that are mediated by enhanced glutamate release. The results indicated that ketamine administration results in increased activity in the mid-posterior cingulate (mid-PCC), thalamus and temporal cortical regions. At the same time, ketamine decreased activity in ventromedial frontal cortex, including orbitofrontal cortex (OFC) and subgenual cingulate (SC), which strongly predicted dissociative effects. Pre-treatment with lamotrigine prevented these effects and many of the BOLD signal changes. The authors concluded that the increase of glutamate release provoked by NMDA receptor blockade results in aberrant perceptual processing in a network of auditory and visual association cortices (centered on the mid-PCC) and in a focal suppression of the OFC-SC and temporal pole, which would prevent the integration of aberrant percepts with visceromotor function and the sense of self. It is worth noting that Deakin et al. (2008) did not directly measure the experience of ego dissolution, and used instead psychiatric rating scales designed to measure dissociative states. Finally, another recent study found that ketamine decreases resting-state functional connectivity of the DMN to the dorsal nexus (a functional node in the bilateral dMPFC), the pregenual anterior cingulate (PACC) and the mPFC via the PCC (Scheidegger et al., 2012). In light of these findings, it is tempting to speculate that aberrant activation of the PCC plays an important role in the occurrence of ego dissolution experiences induced by NDMA receptor antagonists, which would tend to confirm the theory that the neurophysiological basis of DIED is broadly similar across cases involving distinct pharmacological etiologies. While the average item scores on the APZ questionnaire for psilocybin and ketamine reveal that the latter produces stronger feelings of disembodiments, both substances score very high on the OBN subscale of the APZ as well as the “ego consistency” subscale of the EPI questionnaire (Vollenweider and Geyer, 2001; Vollenweider and Kometer, 2010). This broad convergence of the ego dissolving effects of serotoninergic hallucinogens and dissociative anesthetics is confirmed by a recent study with DXM, another NDMA receptor antagonist (Reissig et al., 2012).

### Kappa Opioid Receptor Agonists

The third class of substances seemingly associated with DIED is constituted by KOR agonists such as salvinorin-A, the psychoactive component of the Mexican plant *Salvia divinorum* (Roth et al., 2002). In addition to its primary action as a selective KOR agonist, salvinorin-A has been shown to modify dopamine pathways, decreasing dopamine levels in rats in the caudate putamen (Willmore-Fordham et al., 2007), the dorsal striatum (Gehrke et al., 2008) and the nucleus accumbens (Ebner et al., 2010), thus implicating the dopaminergic reward circuit. It may also interact indirectly with the brain’s endocannabinoid system (Braida et al., 2008). Recreational Salvia users report that the effects of salvironin-A generally differ from those of both serotoninergic hallucinogens and dissociative anesthetics, mainly because they involve less visual distortions, are felt more intensely, and are less enjoyable (Albertson and Grubbs, 2009; Baggott et al., 2010; Addy, 2011). However, there is evidence that salvinorin-A and other selective KOR agonists such as enadoline can induce ego dissolution experiences (Pfeiffer et al., 1986; Walsh et al., 2001; González et al., 2006; Johnson et al., 2011). A recent qualitative analysis of the subjective reports of 30 healthy volunteers found that 37% reported experiencing alterations in their self-awareness with depersonalization-like feelings, and 17% described letting go of their sense of self after the administration of salvinorin-A; 20% also reported a loss of the sense of bodily ownership (Addy et al., 2015). Another recent study suggested that the subjective effects of salvinorin-A, unlike those of serotoninergic hallucinogens, involve intense somatic effects and have a strong dissociative component. At high dosage, it was found to increase scores on the OBN subscale of the APZ questionnaire while decreasing scores on the DED subscale in healthy volunteers (Maqueda et al., 2015). Although qualitative analysis of reports and quantitative analysis with the APZ questionnaire thus converge in suggesting that KOR receptor agonists like Salvia can produce ego dissolution experiences, this ability has yet to be demonstrated with a direct validated measure of DIED, such as the new EDI (Nour et al., 2016). Incidentally, depersonalization disorder might be related to a dysregulation in the opioid system leading to a suppressive effect on stress response (Sierra, 2008), a hypothesis seemingly confirmed by multiple successful trials on depersonalized patients with the opioid antagonist naloxone (Nuller et al., 2001; Simeon and Knutelska, 2005).

Given the lack of brain imagining studies with selective KOR agonists, there is limited knowledge of their effects on regional activity in the brain, let alone how their action on KORs can lead to the subjective occurrence of DIED. KORs are expressed with high density in the striatum, hippocampal dentate gyrus, deep cortical layers V and VI (with more expression in the prefrontal than in the occipital cortex and most densely in the claustrum (Peckys and Landwehrmeyer, 1999). Accordingly, it has been suggested that the subjective effects of Salvia may be due to over-activation of the KOR population in the claustrum, and in particular that the alterations of bodily self-consciousness induced by salvinorin-A might result from the indirect effect of claustrum activation on parietal cortical areas (Stiefel et al., 2014). Further investigations using brain imaging are required to gain a better understanding into the mode of action of selective KOR agonists.

## The Significance of DIED for Cognitive Neuroscience

If DIED is a genuine phenomenon associated with specific neurophysiological mechanisms, what can we expect to learn from its experimental study? It has been argued that if DIED consists in a breakdown of the sense of self, then any neurophysiological change associated with subjective reports of DIED following the administration of psychoactive substances might reveal which functional brain configuration is essential to the maintenance of a sense of self in the baseline state (Carhart-Harris et al., 2012; Lebedev et al., 2015; Nour and Carhart-Harris, 2017). Both the methodological and conceptual components of this program are worth discussing.

### The Search for Neural Correlates of the Sense of Self

The meaning of the correlation between the occurrence of DIED and certain brain states is not entirely unproblematic. There has been considerable debate about methodological issues surrounding the search for the “neural correlates of consciousness” (NCCs) in general. According to an influential definition, an NCC is a minimal neural system N such that there is a mapping from states of N to states of consciousness, where a given state of N is sufficient, under certain enabling conditions, for the corresponding state of consciousness (Chalmers, 2000). These enabling conditions refer to the range of cases in which the neural correlation holds, which may typically include normal brain functioning, unusual inputs and brain stimulation (e.g., transcranial magnetic stimulation), but not important structural or architectural differences, due for instance to brain lesions—which might change the neural correlates of a given conscious state. By analogy, the search for “the neural correlates of the [sense of] self” (Lebedev et al., 2015, pp. 2–3) should be looking for a minimal neural system such that a given state of this system is sufficient, under the relevant enabling conditions, for having a sense of self. However, this is not what the available neurophysiological data on DIED provides. First of all, brain states of subjects undergoing DIED seem to correlate with the *absence* of a sense of self. Thus, the fact that the brain states of DIED subjects show functional differences from those of non-DIED subjects in the same experimental setting (i.e., following the administration of the same dosage of the same psychoactive drug) appears to indicate that these functional differences, or a certain subset of them, account for the loss of the sense of self. In turn, this can be interpreted as evidence that at least some of the neural properties of non-DIED subjects which are changed in DIED cases constitute a necessary condition for the maintenance of a sense of self, at least in normal human brains. However, it is unwarranted to infer from such experimental results that these neural properties are *sufficient* for the maintenance of a sense of self: they might just be neural prerequisites, that is part of the enabling conditions. Furthermore, it is impossible to know which of the functional differences between DIED and non-DIED subjects, if any, directly underlie the loss of the sense of self—that is which of them, if any, is *minimally sufficient* for having a sense of self. For instance, the putative roles of DMN disintegration, oscillatory rhythms in the PCC, salience network integrity and communication between the parahippocampus and the neocortex require further investigation to determine if and how they are linked to the sense of self. In addition, the broad link found in psilocybin and LSD studies between DIED and increased global cortical connectivity/increased entropy does not seem specific enough to warrant any particular claim about the neural correlate of the sense of self. In this regard, the use of psychometrically validated tools to measure DIED, such as the EDI (Nour et al., 2016), might contribute to establish firmer conclusions in the future. In any case, brain imaging studies of DIED can have a more indirect role, by assessing previous hypotheses about the neural correlates of the sense of self, just as imaging of non-conscious processing can cast doubt on hypotheses regarding the neural underpinning of consciousness. As Dehaene et al. (2014, p. 77) put it in a recent article, “if a cognitive computation or neural marker, proposed by some theory to be uniquely associated with conscious processing, can be observed under demonstrably non-conscious conditions, then that theory is severely undermined”. In other words, the search for neural correlates of ego dissolution might indirectly delineate the boundaries of credible hypotheses regarding the neural prerequisites of the sense of self, just as the study of the neural correlates of *unconsciousness* plays a pivotal role in establishing the neurobiological conditions for consciousness (MacDonald et al., 2015).

### The Broad Dichotomy between Two Concepts of Self

In the expression “neural correlates of the (sense of) self”, the word “self” is no less ambiguous than “correlates”. Indeed, the concept of self is extremely polysemous and wide-ranging, and there is no consensus on its meaning in cognitive neuroscience. Attempts to clarify this ambiguity have led to the theorizing of many different notions of the self across a large number of disciplines, including:

*[T]he cognitive self, the conceptual self, the contextualized self, the core self, the dialogic self, the ecological self, the embodied self, the emergent self, the empirical self, the existential self, the extended self, the fictional self, the full-grown self, the interpersonal self, the material self, the narrative self, the philosophical self, the physical self, the private self, the representational self, the rock bottom essential self, the semiotic self, the social self, the transparent self, and the verbal self (Strawson, 2000, p. 39)*.

This lexical proliferation only seems to further obscure the meaning of the search for “neural correlates of the self”, as well as the interpretation of a questionnaire item such as “I experienced a disintegration of my “self” or ego”. It is necessary to determine what definition of the “self” or “ego” is at play in DIED to assess the significance of the phenomenon. Most studies referring to ego dissolution experiences following drug intake describe it as a disruption of the “sense of self”, although there is little agreement on how to understand this notion. Most of the time, it is taken for granted that waking consciousness always involves a sense of self (e.g., “in healthy waking consciousness, one’s sense of self is never far from consciousness”, Carhart-Harris et al., 2014, p. 12), which has been described as “a complex sensory experience of being “me”, as being coherent in mental acts and distinct from the outside world” (Lebedev et al., 2015, p. 2). However, the very notion of a sense of self with a distinctive phenomenology is controversial, and the definition of the kind of “self” which is disrupted in DIED experiences is by no means a trivial matter. Within cognitive neuroscience, there has been a debate in recent years over the interpretation of a host of experimental results, especially regarding the activity of cortical midline structures, purported to give us insight into the neurobiological basis of the “self” (e.g., Qin et al., 2013). In an attempt to clarify this discussion, Legrand and Ruby (2009) have introduced a distinction between self-related and self-specifying processes: while self-related processes underlie tasks in which subjects process information about themselves (such as recognizing themselves or assessing their own personality, physical appearance, attitude or feelings), self-specifying processes implement an implicit self/non-self distinction in perception, action and emotion through sensorimotor feedback loops. They argue that the study of brain processes involved in self-related tasks, such as activity in the mPFC, does not tell us anything important about the neural correlates of the self, since these processes are also involved in inferential processing and memory recall in general (including other-related tasks). By contrast, they suggest that self-specifying processes properly characterize the “subjective perspective”, underpinned by sensorimotor processes relating any represented object to the representing subject. Drawing on a similar idea, a recent article argues that we ought to distinguish between “being a self”, i.e., being a subject of conscious experience with a first-person perspective on the world, and “being [reflectively] self-aware”, i.e., being able to think about oneself as a subject of experience (Musholt, 2013). The author suggests that experimental studies on the self-relatedness of brain processes involving cortical midline nodes focus on reflective self-awareness, whereas psychopathological self-disorders such as those observed in schizophrenia might be more related to the basic aspects of being a self. Both articles agree that the self is not entirely reducible to reflective processes in which one represents oneself, because the condition for this self-representational ability is a more fundamental form of selfhood implemented in sensorimotor processes. In the rich phenomenological tradition, running from the seminal works of Brentano and Husserl at the turn of the 20th century to contemporary philosophy, somewhat similar notions of phenomenal selfhood have been developed. In some of his works, Husserl argued that consciousness always involves a “self-manifestation” (*Für-sich-selbst-erscheinens*), defined as the subjective way in which conscious experience is given (Husserl, 1959, p. 189). Sartre (1943) later introduced the notion of “pre-reflective” self-awareness in *Being and Nothingness*: this minimal kind of self-awareness does not rely on an explicit subject/object distinction; rather, it is “constitutive” of phenomenal consciousness, and is indeed a necessary condition for it. Some contemporary philosophers defend a modern account of pre-reflective self-awareness reminiscent of the Sartrean notion, conceived as the on-going, implicit, first-order awareness of one’s experience as one’s own (Legrand, 2006; Zahavi, 2014). They argue that conscious experience has a distinctive first-personal character, which comes from the fact that it is experienced *as mine*. This aspect of conscious states has also been called “mineness” and “for-me-ness” in recent years (Zahavi and Kriegel, 2016)^3^. According to Legrand and other authors working at the crossroad between phenomenology and cognitive science, this first-personal aspect of experience or pre-reflective self-awareness is what self-specifying processes implement at the subpersonal level (Christoff et al., 2011). It is worth remarking that some phenomenologists who are reluctant to “naturalize” their project are not committed to the assumption that pre-reflective self-awareness can be explained in terms of bodily processes (on this debate see Zahavi, 2004; Kriegel, 2005).

Are those conceptual distinctions illuminating to make sense of DIED experiences? Interestingly (Lebedev et al., 2015, p. 10) proposed to interpret their findings regarding the neuropharmacological mechanisms of DIED by dwelling on another distinction, between the “narrative self” and the “minimal” or “embodied self”. They suggested that the activity of the DMN might be related to the narrative self, while functional brain processes which are affected by DIED might be more specifically related to the minimal/embodied self. The narrative self can be defined as the open-ended construction of one’s own personal identity shaped by social and cultural factors (Gallagher, 2000; Zahavi, 2010), and is associated with high-level cognitive functions such as self-related attitudes, beliefs and autobiographic memory retrieval. The minimal/embodied self (also called “minimal phenomenal selfhood”) refers instead to the basic experience of being a self rooted in bodily sensorimotor processes (Blanke and Metzinger, 2009). Although different authors coming from distinct disciplines and traditions might specify all these distinctions in a slightly different fashion, they appear to be roughly converging towards a general dichotomy between: (a) a basic notion of the self as the first-personal aspect of normal conscious experience implemented by low-level bodily processes; and (b) a more sophisticated conception of the self as constituted by high-level reflective processes involving introspection, self-evaluation and autobiographic memory retrieval. At this stage, it is still an open question whether DIED should be interpreted as a mere disruption of the “narrative self” involving high-level processes linked to personal identity, or as a disruption of the “minimal self” rooted in bodily and sensorimotor processes. Given that self-report of DIED seems typically associated to descriptions of perceptual abnormalities, loss of bodily awareness and disturbance of self-location in space and time, it is tempting to speculate that it is linked to the disruption of low-level perceptual and bodily processes, such as the multisensory integration of self-related stimuli across visual, somatosensory, interoceptive and vestibular domains. However, DIED is a multifaceted experience, and may plausibly disrupt *both* narrative and embodied aspects of selfhood (see Letheby and Gerrans, forthcoming).

Multisensory integration refers to the way in which the processing of stimuli from one sensory modality is sensitive to the information provided by stimuli from another. Information coming from several modalities can thus be integrated to improve perception and solve crossmodal conflicts (Spence and Driver, 2004). The multisensory integration of body-related stimuli has been recently investigated with bodily illusions induced by experimental protocols, such as the rubber hand illusion (Botvinick and Cohen, 1998) and full-body illusions using head-mounted displays (Ehrsson, 2007; Lenggenhager et al., 2007, 2009; Ionta et al., 2011). Along with autoscopic phenomena of neurological origin (including OBEs), full-body illusions induced in experimental settings have been able to dissociate three self-referential components of ordinary conscious experience, namely self-identification with a body (“what is *my* body?”), self-location in space (“where *am I*?”) and the experienced origin of the visuospatial perspective (“from where do *I* experience the world?”) (Blanke and Metzinger, 2009). There is good evidence that these components rely on the multisensory integration of spatiotemporally congruent exteroceptive (mainly visual), somatosensory (tactile and proprioceptive), interoceptive and vestibular signals (Blanke, 2012; Blanke et al., 2015). Multisensory body-related stimuli normally occur within a limited distance from the body, which defines the peripersonal space (PPS). The trunk-centered PPS can be reshaped and extended further away from the body in full-body illusions, which modulates self-identification to a virtual avatar and self-location outside of one’s body (Noel et al., 2015). While body ownership is an integral part of ordinary experience, the idea that self-identification to a physical or hallucinated body is *necessary* for minimal selfhood has been challenged by experiences in which subjects lack the experience of having a body, such as asomatic OBEs and bodiless dreams, which are not typically described as experiences of self-loss. Accordingly, it has been suggested that the simplest form of phenomenal selfhood only requires the experience of spatiotemporal self-location (Windt, 2010, 2015; Metzinger, 2013; see also Limanowski, 2014), which crucially rely on the multisensory integration of visuo-somatosensory and vestibular signals (Pfeiffer et al., 2013). It has been recently proposed that the weakening of self/world boundaries in psychosis might be linked to a disruption of self-location and PPS representation (Noel et al., 2017). Interestingly, phenomenological and neurophysiological evidence regarding DIED seems compatible with a similar hypothesis. Indeed, the experience of unity and the loss of boundaries commonly associated with DIED might be linked to a thorough disruption of the mechanisms underlying normal self-location. Moreover, it has recently been hypothesized that the destabilizing action of serotonergic psychedelics on layer V projections to the thalamus via GABAergic neuronal circuits from sensory areas leads to a disruption of the low-level, spontaneous integration of spatiotemporally congruent multisensory stimuli (Brogaard and Gatzia, 2016). This hypothesis would explain why psychedelic drug intake can lead to both aberrant binding of incongruent exteroceptive stimuli (resulting in synesthesia) and, at high doses, to the breakdown of the integration of congruent visuotactile and vestibular signals underlying self-location (resulting in unitive experiences and DIED). Additionally, recent fMRI studies with full-body illusions suggest that activity in the PCC plays a key role in encoding self-location (Guterstam et al., 2015), alongside multisensory processing in the TPJ (Ionta et al., 2011). Ego dissolution induced by psilocybin and LSD has been associated with decreased oscillatory rhythms in the PCC (Muthukumaraswamy et al., 2013; Carhart-Harris et al., 2014; Lebedev et al., 2016), and NMDA antagonists have been found to decrease activity in the PCC (Olney et al., 1989; Sharp et al., 1991; Yu et al., 2012), while ego dissolution induced by LSD was found to correlate with an increase in TPJ connectivity (Tagliazucchi et al., 2016). This might be an additional evidence that DIED is caused by aberrant multisensory integration of self-related inputs, resulting in a loss of self-location and self/world boundaries. Although more research is needed to assess this proposal, the available data is consistent with the idea that the “sense of self” lost during DIED is not (or not merely) the narrative self, but the minimal self-awareness of ordinary experience rooted in sensorimotor processes. The next section will further examine this hypothesis in light of recent computational models of the minimal/embodied self, with a particular focus on Bayesian models and top-down modulation of multisensory integration.

### DIED and Computational Models of Minimal Selfhood

There is converging evidence that multisensory and motor processing underlies a representation of the self which is continuously updated on the basis of incoming information (Salomon, 2017). Drawing on the distinction between self-related and self-specifying processes, Christoff et al. (2011) have argued that the self/non-self distinction which implicitly specifies the self as an embodied subject and agent is implemented by subpersonal feedback loops, such as those involved in sensorimotor integration and homeostatic regulation. They suggest that the organism continuously distinguishes between “reafference” (afferent signals arising as a result of the organism’s own efferent processes) and “exafference” (afferent signals arising as a result of environmental events), and that the processes underlying this distinction are self-specifying because they implement a unique egocentric perspective in perception and action, which defines the self as the bodily subject and agent of that perspective. On this ground, they further argue that activation of the DMN is neither a necessary condition for self-experience, because activation of the TPN (with a parallel deactivation of the DMN) is not at all incompatible with the experience of being an embodied agent, nor a sufficient condition for self-experience, since regions outside the DMN (such as the lateral PFC and the insula) also seem to play a role in merely self-related processing. In other words, they hold that attention-demanding tasks do not suppress the experience of being an embodied subject and agent underpinned by self-specifying processes, and might even enhance it. This is consistent with the idea that the DMN might underlie narrative rather than embodied aspects of selfhood.

This account of reafference/exafference integration as self-specifying processing fits well within a Bayesian model of the minimal/embodied self. According to the Bayesian brain theory, the brain is a hypothesis testing machine modeling the (hidden) worldly causes of sensory stimuli in a way that approximates Bayesian inference in the long term (Doya et al., 2006). Although many statistical models of bodily multisensory processing have focused on bottom-up integration of stimuli (Ernst and Banks, 2002; Fetsch et al., 2011; Samad et al., 2015), recent research has also emphasized top-down modulation of such integration. The interaction between bottom-up signals and top-down modulation has been modeled by incorporating the free energy principle (FEP) and predictive coding (Friston, 2010; Clark, 2013). According to the FEP, any self-organizing system at equilibrium with its environment must minimize its free energy, thus resisting a natural tendency to disorder implied by the second law of thermodynamics. Applied to neural systems, the FEP means that brain states minimize improbable outcomes (surprise) by minimizing prediction errors through perception and action. This is consistent with the predictive coding framework, according to which the brain generates predictive hierarchical models of the world that are constantly updated on the basis of prediction error signals generated by unexpected sensory information. As mentioned in the previous sections, it has been recently hypothesized that psychedelic substances produce a “disorganized” brain state characterized by a higher neural entropy than the baseline state (Carhart-Harris et al., 2014; Lebedev et al., 2016). In the predictive coding framework, the brain’s predictions crucially depend on top-down prior expectations shaped by experience. In the psychedelic state, however, it has been speculated that the brain’s generative models of the world are under-constrained, and as a result predictions might be suboptimal, which could explain some of the subjective effects of hallucinogenic drugs, such as perceptual distortions, greater cognitive flexibility, greater creativity and feelings of awe and bewilderment with ordinary sensory stimuli (Carhart-Harris et al., 2014). Interestingly, recent theories of the minimal/embodied self converge in suggesting that a Bayesian perspective may contribute to explain how DIED experiences arise. Indeed, several authors have proposed to apply FEP and predictive coding frameworks to develop a neurocomputational model of the embodied self (Carhart-Harris and Friston, 2010; Limanowski and Blankenburg, 2013; Apps and Tsakiris, 2014). In a predictive model of perception, bottom-up sensory error signals are explained away by top-down processes to minimize surprise in multimodal areas further up the hierarchy. It has been suggested that the embodied self ultimately results from the integration of congruent predictions regarding self-related and self-generated (endogenous) multisensory stimuli in high-level supramodal regions (Apps and Tsakiris, 2014). In other words, if the brain predicts the worldly causes of incoming sensory inputs, it also has to predict the organism’s own contribution to those inputs; thus an approximate Bayesian model of the world must include a model of the organism itself or *self-model* (Metzinger, 2003), since this is the most probable explanation of congruent sensorimotor stimuli (Moutoussis et al., 2014; Hohwy and Michael, forthcoming). In terms of the FEP, the hypothesis is that in normal conditions, the model which has the lowest amount of free energy is one in which *the subject itself* is predicted to be the “hidden cause” of congruent somatosensory and homeostatic signals (Limanowski and Blankenburg, 2013). The role of interoceptive signaling in the maintenance of the embodied self has also been emphasized and described within a predictive coding framework (Seth, 2013), and the symptoms of depersonalization disorder have been associated to abnormal interoceptive predictive coding dynamics (Seth et al., 2012). Thus, in normal conditions, the sense of self might result from the generation of a supramodal model of the single cause of congruent multisensory inputs which are “most likely to be me” across exteroceptive, somatosensory, interoceptive and vestibular domains (Apps and Tsakiris, 2014).

This hypothesis could shed light on disturbances of self-experience such as those found in psychosis, or indeed in drug-induced cases. Neurocognitive studies about psychosis converge in indicating that source monitoring deficits and aberrant salience may explain some of the positive symptoms of schizophrenia, and self-disturbances in particular (Nelson et al., 2014a,b). Indeed, if the mechanisms underlying reafferent/exafferent signaling are not functioning properly, this can result in a difficulty to distinguish between the origin of endogenous and exogenous stimuli. For example, if prediction error signals are abnormally encoded for self-generated stimuli, these will be experienced as unpredictable and will not be attenuated, which might eventually lead to the suppression of prediction errors in an updated model where these stimuli are not attributed to the self (Brown et al., 2013). A similar explanation based on predictive coding failures has been put forward to account for the emergence of full-blown delusions from abnormal sensory experiences (Fletcher and Frith, 2009). Likewise, the failure to suppress attention to irrelevant or familiar stimuli in the environment leads to aberrant salience of objects and association, which may be explained by compromised bottom-up and top-down processes underlying predictive perception in psychosis (Keefe et al., 2011). In turn, aberrant salience of self-related stimuli might disturb the continuity and consistency of the embodied self, resulting in alien experiences. Similarly to what happens in psychosis, many subjective effects of hallucinogenic drugs seem to be linked to a failure to make accurate top-down predictions based on contextual information and prior expectancies, resulting in more unconstrained and unreliable perceptual processing, and thus “diminishing confidence or “certainty” about perceived phenomena” (Lebedev et al., 2015, p. 11). Like schizophrenia, hallucinogenic drugs might *improve* the accuracy of participants on a range of tasks in which standard top-down control normally causes a performance decline, as illustrated by a reduced susceptibility to the “hollow mask illusion” (in which healthy subjects see a concave mask as convex face) both in schizophrenia (Emrich, 1989) and after LSD administration (unpublished result of Torsten Passie’s hollow mask study at the Hannover Medical School in Germany). Psilocybin and ayahuasca have also been shown to reduce binocular rivalry switching rate and even allow phenomenal fusion of rival images (Frecska et al., 2003; Carter et al., 2007). Pharmacological models of psychosis emphasize the similar role of disrupted prediction errors signaling both in psychotic and drug-induced cases. The generation of mismatch negativity (MMN), the change in brain activity in response to the occurrence of an unexpected stimulus, is thought to be linked to normal functioning of NMDA receptors (Javitt et al., 1996). A consistent decrease in the amplitude of MMN in response to unexpected stimuli is observed both in schizophrenia patients and in healthy participants pre-treated with ketamine (Corlett et al., 2007; Schmidt et al., 2012). Ketamine and schizophrenia both increase the susceptibility to the rubber hand illusion, resulting in illusory body ownership (Morgan et al., 2011; Thakkar et al., 2011). A recent article has linked the alterations of self-awareness and mind-reading observed in schizophrenic patients to the idea of unstable hierarchical “self-models” in a predictive coding framework, resulting in disrupted self-representation (Nour and Barrera, 2015).

In light of computational models of the minimal/embodied self, it can be hypothesized that ego dissolution is linked to a disruption of top-down and bottom-up processes modulating the multisensory integration of self-related stimuli (across interoceptive, vestibular and visuotactile domains) that normally implement self-location and a self/world boundary. Future research in this area could evaluate whether or not susceptibility to DIED after administration of psychoactive drugs is enhanced by sensory deprivation (which minimizes somatosensory reafferent signals), how psychoactive drugs can affect the experience of full-body illusions using virtual reality (in which self-location is disassociated from self-identification to a body), and if drug intake alters the boundaries of the trunk-centered PPS.

## The Significance of DIED for Philosophy of Mind

While the phenomenon of DIED has not gone unnoticed in neuropharmacological and psychiatric research, it has not yet piqued the interest of philosophers, despite its potential relevance to philosophical debates about selfhood (although see Letheby and Gerrans, forthcoming). Although there is a growing trend in empirically-oriented philosophy of mind to draw upon pathological cases in order to cast doubt on general theoretical claims about perception and cognition (e.g., French, 2015; Macpherson, 2015), non-pathological cases of altered conscious states such as those induced by psychoactive drugs are rarely mentioned. However, there are at least two ways in which the study of DIED could benefit a subset of philosophical debates regarding the self.

### Phenomenal Consciousness and Self-Awareness

The phenomenology of DIED appears to put pressure on the claim that one is self-aware whenever one is conscious. Following the influential formulation of Nagel (1974), a mental state is often said to be phenomenally conscious if and only if there is something it is like for a subject to be in that state. The *qualitative character* of a phenomenally conscious state refers to the experiential qualities or *qualia* of that state: when I have a visual experience of a red apple, “reddishness” is part of the qualitative character of this phenomenally conscious experience. In recent years, several authors have argued that the qualitative character of experience does not exhaust phenomenal consciousness. These authors hold that another aspect of conscious experience exists alongside qualia, namely its *subjective character* (Levine, 2001; Kriegel, 2005). The subjective character of a conscious state refers to the fact that there is something it is like *for me* to be in a given state. As some of the advocates of this distinction put it, the subjective character does not capture the *what-it-is-like-ness* of the conscious state, but its *for-me-ness*: while my experiences of seeing a red apple and seeing a green apple differ in their qualitative character, they are said to be the same with respect to their subjective character, because in both cases it is *for me* that it is like something to have them (Zahavi and Kriegel, 2016). According to a bundle of theories which have been collectively referred to as “subjectivity theories of consciousness”, subjective character is a necessary condition of phenomenal consciousness, because the two are constitutively linked (Billon and Kriegel, 2015)^4^. These theories endorse the following “subjective character principle” (SCP):

*(SCP) Necessarily, a mental state is phenomenally conscious if and only if it has subjective character*.

However, the notion of subjective character is ambiguous (see Guillot, 2017): while it is sometimes defined as an “inner” or “peripheral” awareness of one’s own experience, it has also been equated with a kind of self-awareness (e.g., Zahavi, 2014). Therefore, it is not clear that so-called subjectivity theories all endorse the claim that self-awareness properly speaking (an awareness *of oneself*, or a sense of self) is a necessary condition for consciousness. In any case, even outside of the debate about SCP, many publications in neuroscience, psychology and philosophy have endorsed the general idea that consciousness necessarily involves some kind of sense of self or self-awareness (e.g., Damasio, 1999; Gallagher, 2000; Gallagher and Zahavi, 2005; Strawson, 2011; Lou et al., 2016). Let us call this specific claim the “self-awareness principle” (SAP)^5^:

*(SAP) Necessarily, whenever one is in a conscious state, one is minimally self-aware*.

This is a theoretical claim, but one that ultimately bears on empirical matters: if it were shown that there are in fact instances in which a subject is in a phenomenally conscious state without being self-aware in any way, then SAP would be *prima facie* invalidated on empirical grounds. For instance, Kriegel admits that he “cannot envisage what it would be like to have a phenomenology lacking the kind of inner awareness that constitutes for-me-ness.” (Kriegel, 2009, p. 175)^6^. However, a number of pathological cases have been brought up as potential threats to such a claim. Billon and Kriegel (2015) have discussed some of these anomalous experiences under the label “alienation symptoms”, including thought insertion, delusions of alien control, somatoparaphrenia and loss of bodily ownership. According to them, alienation symptoms can be explained within subjectivity theories, if they arise when a first order non-conscious alien mental state occurs in simultaneity with a second order non-alien conscious state that represents the first order state. It has been argued that this explanation is inconsistent with empirical data about the neural correlates of alienation experiences (Lane, 2015), but regardless of this issue it is not clear that it can properly accommodate the phenomenon of DIED. If I have a thought that feels inserted in my mind, it still makes sense to say that *I* am aware of this thought, and indeed that *I* am aware of it as alien. However, subjective reports of ego dissolution experiences suggest that they lack this first-personal aspect altogether. In narrative self-reports, drug users are often reluctant to use the first person pronoun at all when describing DIED. For instance, one narrative report describing a DIED experience after intake of *Psilocybe* mushrooms includes the following comment: “There existed no one, not even me … so would it be proper to still speak of “I”, even as the notion of “I” seemed so palpably illusory?”^7^. Nevertheless, self-reports clearly converge in indicating that DIED is a conscious experience, and one that is memorable.

### The Phenomenology of Minimal Selfhood

The proper assessment of SAP crucially depends on whether there is indeed a kind of minimal self-awareness or sense of self surfacing at the personal level in conscious experience, rather than merely subpersonal self-specifying processes with no counterpart at the conscious level. If so, it is presumably not a salient feature of experience, but rather something like a structural “invariant” that goes unnoticed most of the time^8^. But even on such a reading, DIED experiences seem to threaten SAP. While the sense of self might be in the background of normal waking consciousness in such a way that we don’t usually pay attention to (or focus on) this aspect of experience, ego dissolution phenomena suggest that such background awareness can suddenly become prominent when it is deeply altered—somewhat as the interruption of background music suddenly brings to one’s focal attention the fact that it was discreetly playing before. Thus, DIED experiences present an interest for philosophers as a potential challenge to the idea that there is no phenomenology of the self. Even if it is true, as Hume famously wrote in his *Treatise of Human Nature*, that the “self” is nowhere to be found when I survey the contents of my conscious experience, the fact that DIED is described as a breakdown of the alleged subjective structure of experience may indicate that such structure is otherwise part of one’s conscious experience. Reminiscent of how endogenous stimuli such as self-produced tactile stimulation are attenuated in normal conditions, minimal phenomenal selfhood need not be a salient feature of one’s conscious states. According to the hypothesis presented in this article, it might be implicit in conscious experience when multisensory endogenous stimuli are correctly integrated at the subpersonal level, and might only come to the fore when these self-specifying processes start dysfunctioning. Therefore, philosophers interested in self-awareness and minimal/embodied selfhood might draw on descriptions of DIED experiences to discuss the extent to which a minimal sense of self is indeed part of one’s conscious experience in normal situations. There is room, however, for at least two different interpretations of DIED which do not threaten SAP. The first alternative interpretation, mentioned in the section “The Broad Dichotomy between Two Concepts of Self”, takes DIED to be a mere disruption of *high-level* processes underlying the “narrative self” (such as one’s network of self-related beliefs and autobiographical memory). On this construal, DIED might impair “reflective” or “conceptual” self-consciousness (one’s ability to think about oneself and self-ascribe conscious experiences) without undermining the subjective character of consciousness. According to a second alternative interpretation, DIED does not *subtract* something that was supposedly present before in the experience, but rather *adds* something to the ordinary structure of consciousness, such as a feeling of alienation. As mentioned in the section “Phenomenal Consciousness and Self-Awareness”, this “something extra” strategy has been invoked by proponents of subjectivity theories to deny that thought insertion and passivity experiences could threaten their claim (Billon and Kriegel, 2015; Zahavi and Kriegel, 2016). It is worth noting that a similar strategy can also be used by opponents of SAP; indeed, it may be that the subpersonal processes disrupted by DIED do not normally underlie any kind of self-awareness, while their disruption does produce the feeling of losing one’s sense of self. If this was the case, then self-specifying processes such as multisensory processing of self-related stimuli would not normally correlate with any kind of “sense of self”, but their disturbance would correlate with the experience of ego dissolution. Although this interpretation is not excluded by available evidence, it does not sit very well with the hypothesis that the processes undermined during DIED are linked to bodily awareness and the experience of spatiotemporal self-location, since there is some empirical evidence that the latter does pervade ordinary conscious experience (Blanke and Metzinger, 2009; Bermúdez, 2011; Serino et al., 2013; Maselli, 2015; Huang et al., 2017). According to another alternative interpretation, it may be the case that DIED alters self-awareness through a dissociation of automatic processing from the self, resulting in the experience of perception, thought and movement as alien. Such a hypothesis is rooted in the “perceptual anomalies” approach to schizophrenic self-disturbances in schizophrenia, inspired by Beringer’s and Mayer-Gross’ early experiments with mescaline (Beringer, 1927; Sterzer et al., 2016).

## General Summary and Future Directions

This article reviewed current knowledge about the phenomenon known as DIED. While there is good evidence from both anecdotal narrative reports and quantitative data that the experience of losing one’s sense of self can occur in healthy subjects following the intake of certain psychoactive drugs at high doses, its neurophysiological etiology is not yet fully understood. This experience seems to occur with three neuropharmacological classes of substances: classical psychedelics which act as selective agonists of the serotonin 2A receptor subtype, dissociative anesthetics which act as antagonists of the glutamate NDMA receptor subtype, and selective KOR agonists. Whether or not the neurocognitive mechanisms which bring about DIED experiences are broadly similar with these three classes of substances, despite their primary action on different neurotransmitter receptors, remains an open question. The occurrence of DIED after psychedelic intake (psilocybin and LSD) appears to correlate with DMN disintegration, increased global functional connectivity and increased neural entropy, but further research is required to establish more fine-grained correlations and compare the results with other classes of psychoactive drugs. The dependence of DIED experiences on doses could also be better understood. In addition, more research is needed to assess the phenomenological unity of DIED across compounds. One promising avenue of investigation is the use of specific validated questionnaires such as the EDI for a systematic assessment of the extent to which each psychoactive compound can produce ego dissolution. There is now good evidence in the case of classical psychedelics that DIED is a valid and unidimensional construct which can be reliably measured (Nour et al., 2016). However, there are also well-documented differences between the subjective effects of classical psychedelics, dissociative anesthetics and KOR agonists (e.g., geometrical patterns with psilocybin and LSD, experiences of disembodiment with ketamine, or interoceptive and somatosensory illusions with Salvia). It is intriguing to speculate that ego dissolution does not share the exact same experiential characteristics across these different classes of compounds, although more evidence is required. More generally, one may wonder to what extent experiences of ego dissolution induced by drugs, psychosis and deep meditation share a common core phenomenology.

DIED is often described as the loss of one’s “sense of self”, and narrative reports converge in suggesting that it is indeed experienced as the breakdown of the basic subjective character of conscious experience. Recent neurocomputational models of the “minimal” or “embodied” self, which refers to the experience of being a subject in perception and action, suggest that it might be related to multisensory processing of self-related stimuli, and explained within Bayesian models (Limanowski and Blankenburg, 2013). In such models, minimal phenomenal selfhood relies on the proper integration of congruent visual, somatosensory, interoceptive and vestibular feedback about one’s body, location and orientation in higher cortical areas, which specifies a minimal sense of self as the result of hierarchical predictions minimizing surprise (i.e., explaining away prediction errors). Psychoactive drugs able to induce ego dissolution have been hypothesized to disrupt the weighting of top-down predictions and bottom-up prediction errors in perception, including multisensory processing. Therefore, such computational models of phenomenal selfhood are consistent with the occurrence of DIED experiences, and might even be good candidates to explain them. Moreover, similar computational mechanisms have been hypothesized to underpin disturbances of self-experience in acute psychosis. This theoretical convergence informs the hypothesis that DIED is linked to abnormal processing of multisensory endogenous stimuli, leading to the loss of self/world boundary and self-location, and eventually feelings of non-existence. DIED experiences are also relevant to philosophical discussions about the subjective character of experience, insofar as they challenge the claim that minimal self-awareness is necessary for consciousness. On the other hand, they also seem consistent with the idea that *ordinary* conscious experience involves a minimal kind of self-awareness, characterized by self-referential aspects such as body ownership, self-location and the egocentric structure of the visuospatial perspective. This might explain why the occurrence of DIED, in which these aspects appear to be missing (or at least thoroughly altered), is perceived as such a dramatic change in the structure of conscious experience.

Given the relevance of DIED to a number of theoretical issues in both cognitive science and philosophy, it is important to gain a better understanding of its phenomenology, beyond the limited level of detail usually found in psychometric questionnaires and online narrative self-reports. Future research could greatly benefit from fined-grained descriptions of the experience from onset to end, using proven methods to avoid theoretical and cognitive biases. For instance, the micro-phenomenological technique developed by Vermersch and Petitmengin was devised to elicit a disciplined description of the past lived experience associated with a given cognitive process, including subtle aspects unnoticed by the subject at first (Petitmengin, 2006; Vermersch and Pierre, 1994). This method has proven particularly efficient for eliciting very detailed descriptions of “the micro-dynamics of experience” at the level of brief mental events (Petitmengin and Lachaux, 2013). It has been independently applied to anomalous bodily experiences such as the rubber hand illusion (Valenzuela Moguillansky et al., 2013) and the loss of self/world boundaries in expert mindfulness meditation (Ataria et al., 2015; Petitmengin et al., forthcoming). The use of similar techniques with DIED subjects in a controlled setting could overcome the issue of the seemingly “ineffable” character of the experience, and bring to light fine-grained aspects of the phenomenon which have not yet been well documented^9^. First-person methods could also be incorporated within neurocognitive studies, in the spirit of “neurophenomenology” (Varela, 1996; Gallagher, 2003), to further bridge the gap between subjective accounts and brain imaging of DIED, and find more detailed neural correlations. Ego dissolution experiences are so remote from normal waking consciousness that it is important for researchers to rely on detailed and reliable subjective descriptions that can be cross-validated. The complementary use of both quantitative methods (such as the new EDI) and qualitative methods (such as the microphenomenological interview) could go a long way towards establishing a science of DIED.

## Author Contributions

RM is the only contributor of this article.

## Funding

The publication of this article was generously supported by the Faculty of Philosophy of the University of Oxford and Magdalen College.

## Conflict of Interest Statement

The author declares that the research was conducted in the absence of any commercial or financial relationships that could be construed as a potential conflict of interest.

## Footnotes

^1^While the expression “ego death” became widely known in the 60s counterculture among drug users in the wake of Timothy Leary’s influential publications on the psychedelic experience, the scientific literature has privileged “ego dissolution” in recent years (e.g., Lebedev et al., 2015; Nour et al., 2016). “Ego disintegration” has also been used in a few recent studies (Muthukumaraswamy et al., 2013; Carhart-Harris et al., 2014).

^2^http://www.erowid.org

^3^It is worth noting that the historical roots and conceptual soundness of the notions of pre-reflective self-awareness and mineness have been debated, even within the phenomenological tradition, with respect to the study of self-disturbances in schizophrenia (Mishara, 2007; Sass et al., 2011; Mishara et al., 2014; Grosz, 2015). Additionally, there is not always a clear distinction in the literature between awareness of one’s experience and awareness of oneself (see Guillot, 2017).

^4^According to Billon and Kriegel, subjectivity theories include acquaintance theories (Levine, 2001), higher-order representationalism (Gennaro, 2012) and self-representationalism (Kriegel, 2009).

^5^Most proponents of SCP seem to accept SAP (e.g., Zahavi, 2005; Kriegel, 2009, p. 177), and some even claim that the latter is entailed by the former (see Rosenthal, 1997, p. 741).

^6^Although Kriegel seems to define for-me-ness as “peripheral inner awareness” of one’s concurrent experience here, he defines it elsewhere as “a minimum point of self-consciousness” (Zahavi and Kriegel, 2016, p. 49). He comes close to explicitly endorsing SAP, although he does not formulate a necessity claim: “my current experience’s pre-reflective self-consciousness strikes me as egological—that is, as a form of peripheral *self*-awareness… There is an elusive sense of self-presence or self-manifestation inherent in even a simple conscious experience of the blue sky.” (Kriegel, 2009; p. 177). See also Damasio (1999, p. 19): “If ‘self-consciousness’ is taken to mean ‘consciousness with a sense of self’, then all human consciousness is necessarily covered by the term—there is just no other kind of consciousness as far as I can see”; and Lou et al. (2016): “self-awareness is an integral function of all conscious experiences”.

^7^https://erowid.org/experiences/exp.php?ID=42740

^8^Note that this hypothesis is not committed to the controversial claim that attention is not necessary for consciousness (Mole, 2008). For instance, minimal self-awareness could be modulated by *diffuse* rather than *focal* attention (Prettyman, 2014). On some readings however, it might be committed to a moderate “abundance view” of consciousness (Schwitzgebel, 2007; see Alsmith, 2012).

^9^It should be mentioned that such efforts have historical precedents in the rigorous work of the early Heidelberg School and other clinical phenomenologists, e.g., Binswanger, Straus, Minkowski, and Conrad.

## References

[B1] Addy P. H. (2011). Acute and post-acute behavioral and psychological effects of salvinorin a in humans. Psychopharmacology (Berl) 220, 195–204. 10.1007/s00213-011-2470-6 21901316

[B2] Addy P. H. Garcia-Romeu A. Metzger M. Wade J. (2015). The subjective experience of acute, experimentally-induced salvia divinorum inebriation. J. Psychopharmacol. 29, 426–435. 10.1177/0269881115570081 25691501

[B3] Albertson D. N. Grubbs L. E. (2009). Subjective effects of salvia divinorum: LSD- or marijuana-like? J. Psychoactive Drugs 41, 213–217. 10.1080/02791072.2009.10400531 19999674

[B4] Alnæs R. (1964). Therapeutic application of the change in consciousness produced Ry psycholytica (LSD, psilocybin, Etc.): the psychedelic experience in the treatment of neurosis. Acta Psychiatr. Scand. 39, 397–409. 10.1111/j.1600-0447.1964.tb04952.x 14345225

[B5] Alonso J. F. Romero S. Mañanas M. À. Riba J. (2015). Serotonergic psychedelics temporarily modify information transfer in humans. Int. J. Neuropsychopharmacol. 18:pyv039. 10.1093/ijnp/pyv039 25820842 PMC4571623

[B6] Alsmith A. (2012). “What reason could there be to believe in pre-reflective bodily self-consciousness,” in Consciousness in Interaction: The Role of the Natural and Social Environment in Shaping Consciousness, ed. Paglieri F. (Amsterdam: John Benjamins Press), 1–18.

[B7] Anand A. Charney D. S. Oren D. A. Berman R. M. Hu X. S. Cappiello A.. (2000). Attenuation of the neuropsychiatric effects of ketamine with lamotrigine: support for hyperglutamatergic effects of *N-* methyl-D-aspartate receptor antagonists. Arch. Gen. Psychiatry 57, 270–276. 10.1001/archpsyc.57.3.270 10711913

[B8] Anderson E. W. Rawnsley K. (1954). Clinical studies of lysergic acid diethylamide. Monatsschr. Psychiatr. Neurol. 128, 38–55. 10.1159/000139775 13203446

[B9] Anis N. A. Berry S. C. Burton N. R. Lodge D. (1983). The dissociative anaesthetics, ketamine and phencyclidine, selectively reduce excitation of central mammalian neurones by N-methyl-aspartate. Br. J. Pharmacol. 79, 565–575. 10.1111/j.1476-5381.1983.tb11031.x 6317114 PMC2044888

[B10] Apps M. A. Tsakiris M. (2014). The free-energy self: a predictive coding account of self-recognition. Neurosci. Biobehav. Rev. 41, 85–97. 10.1016/j.neubiorev.2013.01.029 23416066 PMC3848896

[B11] Aspell J. E. Heydrich L. Marillier G. Lavanchy T. Herbelin B. Blanke O. (2013). Turning body and self inside out visualized heartbeats alter bodily self-consciousness and tactile perception. Psychol. Sci. 24, 2445–2453. 10.1177/0956797613498395 24104506

[B12] Ataria Y. Dor-Ziderman Y. Berkovich-Ohana A. (2015). How does it feel to lack a sense of boundaries? A case study of a long-term mindfulness meditator. Conscious. Cogn. 37, 133–147. 10.1016/j.concog.2015.09.002 26379087

[B13] Baggott M. J. Erowid E. Erowid F. Galloway G. P. Mendelson J. (2010). Use patterns and self-reported effects of salvia divinorum: an internet-based survey. Drug Alcohol Depend. 111, 250–256. 10.1016/j.drugalcdep.2010.05.003 20627425

[B14] Bartolomei F. Barbeau E. J. Nguyen T. McGonigal A. Régis J. Chauvel P.. (2012). Rhinal-hippocampal interactions during Déjà vu. Clin. Neurophysiol. 123, 489–495. 10.1016/j.clinph.2011.08.012 21924679

[B15] Baumeister R. F. Exline J. J. (2002). Mystical self loss: a challenge for psychological theory. Int. J. Psychol. Relig. 12, 15–20. 10.1207/s15327582ijpr1201_02

[B16] Bente D. Itil T. Schmid E. E. (1958). [EEG findings on the action mechanism of LSD 25]. Psychiatr. Neurol. (Basel) 135, 273–284. 10.1159/000131920 13554709

[B17] Bercel N. A. Travis L. E. Olinger L. B. Dreikurs E. Polos M. G. (1956). Model psychoses induced by LSD-25 in normals: I. psychophysiological investigations, with special reference to the mechanism of the paranoid reaction. AMA Arch. Neurol. Psychiatry 75, 588–611. 10.1001/archneurpsyc.1956.02330240026003 13325989

[B18] Beringer K. (1927). Der Meskalinrausch: Seine Geschichte Und Erscheinungsweise. Monographie Neurol Psychiatry H (49) Available online at: https://books.google.co.uk/books?hl=fr&lr=&id=bA2zBgAAQBAJ&oi=fnd&pg=PA1&dq=beringer+der+meskalinrauschandots=xLVNbyrL1p&sig=J-sxKn5spyiFzggHTAc0x4y9i24

[B19] Bermúdez J. L. (2011). “Bodily awareness and self-consciousness,” in The Oxford Handbook of the Self, ed. Gallagher S. (Oxford, UK: Oxford University Press), 157–179.

[B20] Billon A. Kriegel U. (2015). “Jaspers’ dilemma: the psychopathological challenge to subjectivity theories of consciousness,” in Disturbed Consciousness: New Essays on Psychopathology and Theories of Consciousness, ed. Gennaro R. J., (Cambridge, MA: MIT Press), 29–54.

[B21] Blanke O. (2012). Multisensory brain mechanisms of bodily self-consciousness. Nat. Rev. Neurosci. 13, 556–571. 10.1038/nrn3292 22805909

[B22] Blanke O. Arzy S. (2005). The out-of-body experience: disturbed self-processing at the temporo-parietal junction. Neuroscientist 11, 16–24. 10.1177/1073858404270885 15632275

[B23] Blanke O. Metzinger T. (2009). Full-body illusions and minimal phenomenal selfhood. Trends Cogn. Sci. 13, 7–13. 10.1016/j.tics.2008.10.003 19058991

[B24] Blanke O. Slater M. Serino A. (2015). Behavioral, neural and computational principles of bodily self-consciousness. Neuron 88, 145–166. 10.1016/j.neuron.2015.09.029 26447578

[B25] Blei D. M. Ng A. Y. Jordan M. I. (2003). Latent dirichlet allocation. J. Mach. Learn. Res. 3, 993–1022.

[B26] Bodmer I. Dittrich A. Lamparter D. (1994). Aussergewöhnliche bewusstseinszustände—ihre gemeinsame struktur und messung. Welten Des Bewus Stseins 3, 45–58.

[B27] Botvinick M. Cohen J. (1998). Rubber hands ‘feel’ touch that eyes see. Nature 391:756. 10.1038/35784 9486643

[B28] Bowers M. B. Freedman D. X. (1966). “Psychedelic” experiences in acute psychoses. Arch. Gen. Psychiatry 15, 240–248. 10.1001/archpsyc.1966.01730150016003 5911238

[B29] Braida D. Limonta V. Capurro V. Fadda P. Rubino T. Mascia P.. (2008). Involvement of κ-Opioid and endocannabinoid system on salvinorin a-induced reward. Biol. Psychiatry 63, 286–292. 10.1016/j.biopsych.2007.07.020 17920565

[B30] Brefczynski-Lewis J. A. Lutz A. Schaefer H. S. Levinson D. B. Davidson R. J. (2007). Neural correlates of attentional expertise in long-term meditation practitioners. Proc. Natl. Acad. Sci. U S A 104, 11483–11488. 10.1073/pnas.0606552104 17596341 PMC1903340

[B31] Bremmer F. Klam F. Duhamel J. R. Ben Hamed S. Graf W. (2002). Visual-vestibular interactive responses in the macaque ventral intraparietal area (VIP). Eur. J. Neurosci. 16, 1569–1586. 10.1046/j.1460-9568.2002.02206.x 12405971

[B32] Brewer J. A. Garrison K. A. Whitfield-Gabrieli S. (2013). What about the ‘self’ is processed in the posterior cingulate cortex? Front. Hum. Neurosci. 7:647. 10.3389/fnhum.2013.00647 24106472 PMC3788347

[B33] Brewer J. A. Worhunsky P. D. Gray J. R. Tang Y. Y. Weber J. Kober H. (2011). Meditation experience is associated with differences in default mode network activity and connectivity. Proc. Natl. Acad. Sci. U S A 108, 20254–20259. 10.1073/pnas.1112029108 22114193 PMC3250176

[B34] Brogaard B. Gatzia D. E. (2016). “Psilocybin, lysergic acid diethylamide, mescaline, and drug-induced synesthesia,” in Neuropathology of Drug Addictions and Substance Misuse (Vol. 2), ed. Preedy V. R. (Cambridge, MA: Academic Press), 890–905.

[B35] Brown H. Adams R. A. Parees I. Edwards M. Friston K. (2013). Active inference, sensory attenuation and illusions. Cogn. Process. 14, 411–427. 10.1007/s10339-013-0571-3 23744445 PMC3824582

[B36] Buckner R. L. Andrews-Hanna J. R. Schacter D. L. (2008). The brain’s default network: anatomy, function and relevance to disease. Ann. N Y Acad. Sci. 1124, 1–38. 10.1196/annals.1440.011 18400922

[B37] Bürgy M. (2011). Ego disturbances in the sense of kurt schneider: historical and phenomenological aspects. Psychopathology 44, 320–328. 10.1159/000325059 21734435

[B38] Calvert G. A. Campbell R. Brammer M. J. (2000). Evidence from functional magnetic resonance imaging of crossmodal binding in the human heteromodal cortex. Curr. Biol. 10, 649–657. 10.1016/s0960-9822(00)00513-3 10837246

[B39] Carhart-Harris R. L. Erritzoe D. Williams T. Stone J. M. Reed L. J. Colasanti A.. (2012). Neural correlates of the psychedelic state as determined by fMRI studies with psilocybin. Proc. Natl. Acad. Sci. U S A 109, 2138–2143. 10.1073/pnas.1119598109 22308440 PMC3277566

[B40] Carhart-Harris R. L. Friston K. J. (2010). The default-mode, ego-functions and free-energy: a neurobiological account of freudian ideas. Brain 133, 1265–1283. 10.1093/brain/awq010 20194141 PMC2850580

[B41] Carhart-Harris R. L. Leech R. Erritzoe D. Williams T. M. Stone J. M. Evans J.. (2013). Functional connectivity measures after psilocybin inform a novel hypothesis of early psychosis. Schizophr. Bull. 39, 1343–1351. 10.1093/schbul/sbs117 23044373 PMC3796071

[B42] Carhart-Harris R. L. Leech R. Hellyer P. J. Shanahan M. Feilding A. Tagliazucchi E.. (2014). The entropic brain: a theory of conscious states informed by neuroimaging research with psychedelic drugs. Front. Hum. Neurosci. 8:20. 10.3389/fnhum.2014.00020 24550805 PMC3909994

[B43] Carhart-Harris R. L. Muthukumaraswamy S. Roseman L. Kaelen M. Droog W. Murphy K.. (2016). Neural correlates of the lsd experience revealed by multimodal neuroimaging. Proc. Natl. Acad. Sci. U S A 113, 4853–4858. 10.1073/pnas.1518377113 27071089 PMC4855588

[B44] Carter O. L. Hasler F. Pettigrew J. D. Wallis G. M. Liu G. B. Vollenweider F. X. (2007). Psilocybin links binocular rivalry switch rate to attention and subjective arousal levels in humans. Psychopharmacology (Berl) 195, 415–424. 10.1007/s00213-007-0930-9 17874073

[B45] Chalmers D. J. (2000). “What is a neural correlate of consciousness?,” in Neural Correlates of Consciousness, ed. Metzinger T. (Cambridge, MA: MIT Press), 17–39.

[B46] Chambers R. A. Bremner J. D. Moghaddam B. Southwick S. M. Charney D. S. Krystal J. H. (1999). Glutamate and post-traumatic stress disorder: toward a psychobiology of dissociation. Semin. Clin. Neuropsychiatry 4, 274–281. 10553033 10.153/SCNP00400274

[B47] Christoff K. Cosmelli D. Legrand D. Thompson E. (2011). Specifying the self for cognitive neuroscience. Trends Cogn. Sci. 15, 104–112. 10.1016/j.tics.2011.01.001 21288760

[B48] Clark A. (2013). Whatever next? predictive brains, situated agents, and the future of cognitive science. Behav. Brain Sci. 36, 181–204. 10.1017/S0140525x12000477 23663408

[B49] Cohen S. (1960). Lysergic acid diethylamide: side effects and complications. J. Nerv. Ment. Dis. 130, 30–40. 10.1097/00005053-196001000-00005 13811003

[B50] Corlett P. R. Honey G. D. Fletcher P. C. (2007). From prediction error to psychosis: ketamine as a pharmacological model of delusions. J. Psychopharmacol. 21, 238–252. 10.1177/0269881107077716 17591652

[B51] Coyle J. R. Presti D. E. Baggott M. J. (2012). Quantitative analysis of narrative reports of psychedelic drugs. arXiv:1206.0312 [Q-Bio]. Available Online at: http://arxiv.org/abs/1206.0312.

[B52] Craig A. D. (2009). How do you feel—now? The anterior insula and human awareness. Nat. Rev. Neurosci. 10, 59–70. 10.1038/nrn2555 19096369

[B53] Cummins C. Lyke J. (2013). Peak experiences of psilocybin users and non-users. J. Psychoactive Drugs 45, 189–194. 10.1080/02791072.2013.785855 23909006

[B54] Damasio A. (1999). The Feeling of What Happens: Body and Emotion in the Making of Consciousness. New York, NY: Houghton Mifflin Harcourt.

[B55] De Simoni S. Schwarz A. J. O’Daly O. G. Marquand A. F. Brittain C. Gonzales C.. (2013). Test-retest reliability of the BOLD pharmacological MRI response to ketamine in healthy volunteers. Neuroimage 64, 75–90. 10.1016/j.neuroimage.2012.09.037 23009959

[B56] Deakin J. F. Lees J. McKie S. Hallak J. E. Williams S. R. Dursun S. M. (2008). Glutamate and the neural basis of the subjective effects of ketamine: a pharmaco-magnetic resonance imaging study. Arch. Gen. Psychiatry 65, 154–164. 10.1001/archgenpsychiatry.2007.37 18250253

[B57] Dehaene S. Charles L. King J. R. Marti S. (2014). Toward a computational theory of conscious processing. Curr. Opin. Neurobiol. 25, 76–84. 10.1016/j.conb.2013.12.005 24709604 PMC5635963

[B58] Denber H. C. B. (1958). Studies on mescaline viii: psychodynamic observations. Am. J. Psychiatry 115, 239–244. 10.1176/ajp.115.3.239 13571442

[B60] Dittrich A. (1975). Zusammenstellung Eines Fragebogens (APZ) Zur Erfassung abnormer psychischer Zustände [Construction of a questionnaire (APZ) for assessing abnormal mental states]. Z. Klin Psychol. Psychiatr. Psychother. 23, 12–20.

[B61] Dittrich A. (1996). Ätiologie-Unabhängige Strukturen Veränderter Wachbewusstseinszustände. Ergebnisse Empirischer Untersuchungen Über Halluzinogene I. Und II. Ordnung, Sensorische Deprivation, Hypnagoge Zustände, Hypnotische Verfahren Sowie Reizüberflutung. Berlin: Verlag für Wissenschaft und Bildung.

[B62] Dittrich A. (1998). The standardized psychometric assessment of altered states of consciousness (ASCs) in humans. Pharmacopsychiatry 31, 80–84. 10.1055/s-2007-979351 9754838

[B63] Dittrich A. Lamparter D. Maurer M. (2006). 5D-ABZ: Fragebogen Zur Erfassung Aussergewöhnlicher Bewusstseinszustände. Eine Kurze Einführung. Zürich: PSIN Plus.

[B59] Dittrich A. Lamparter D. Maurer M. (2010). 5D-ASC: Questionnaire for the Assessment of Altered States of Consciousness. A Short Introduction. Zurich: PSIN PLUS.

[B64] Dor-Ziderman Y. Berkovich-Ohana A. Glicksohn J. Goldstein A. (2013). Mindfulness-induced selflessness: a MEG neurophenomenological study. Front. Hum. Neurosci. 7:582. 10.3389/fnhum.2013.00582 24068990 PMC3781350

[B65] Doya K. Ishii S. Pouget A. Rao R. P. N. (Eds.) (2006). Bayesian Brain: Probabilistic Approaches to Neural Coding. 1st Edn. Cambridge, MA: The MIT Press.

[B66] Ebner S. R. Roitman M. F. Potter D. N. Rachlin A. B. Chartoff E. H. (2010). Depressive-like effects of the kappa opioid receptor agonist salvinorin A are associated with decreased phasic dopamine release in the nucleus accumbens. Psychopharmacology (Berl) 210, 241–252. 10.1007/s00213-010-1836-5 20372879 PMC2894632

[B67] Eddy C. M. (2016). The junction between self and other? Temporo-parietal dysfunction in neuropsychiatry. Neuropsychologia 89, 465–477. 10.1016/j.neuropsychologia.2016.07.030 27457686

[B68] Ehrsson H. H. (2007). The experimental induction of out-of-body experiences. Science 317:1048. 10.1126/science.1142175 17717177

[B69] Eisner B. G. Cohen S. (1958). Psychotherapy with lysergic acid diethylamide. J. Nerv. Ment. Dis. 127, 528–539. 10.1097/00005053-195812000-00006 13621221

[B70] Emrich H. M. (1989). A three-component-system hypothesis of psychosis. Impairment of binocular depth inversion as an indicator of a functional dysequilibrium. Br. J. Psychiatry 37–39. 2690888

[B71] Ernst M. O. Banks M. S. (2002). Humans integrate visual and haptic information in a statistically optimal fashion. Nature 415, 429–433. 10.1038/415429a 11807554

[B72] Fetsch C. R. Pouget A. DeAngelis G. C. Angelaki D. E. (2011). Neural correlates of reliability-based cue weighting during multisensory integration. Nat. Neurosci. 15, 146–154. 10.1038/nn.2983 22101645 PMC3398428

[B73] Fletcher P. C. Frith C. D. (2009). Perceiving is believing: a bayesian approach to explaining the positive symptoms of schizophrenia. Nat. Rev. Neurosci. 10, 48–58. 10.1038/nrn2536 19050712

[B74] Frecska E. White K. D. Luna L. E. (2003). Effects of the amazonian psychoactive beverage ayahuasca on binocular rivalry: interhemispheric switching or interhemispheric fusion? J. Psychoactive Drugs 35, 367–374. 10.1080/02791072.2003.10400019 14621134

[B75] French C. (2015). Bálint’s syndrome and the structure of visual experience. Unpublished Manuscript. Available Online at: http://craigafrench.github.io/assets/CFBalintsPaper.pdf.

[B76] Friston K. (2010). The free-energy principle: a unified brain theory? Nat. Rev. Neurosci. 11, 127–138. 10.1038/nrn2787 20068583

[B78] Gallagher S. (2000). Philosophical conceptions of the self: implications for cognitive science. Trends Cogn. Sci. 4, 14–21. 10.1016/s1364-6613(99)01417-5 10637618

[B79] Gallagher S. (2003). Phenomenology and experimental design: toward a phenomenologically enlightened experimental science. J. Conscious. Stud. 10, 85–99.

[B77] Gallagher S. Zahavi D. (2005). Phenomenological approaches to self-consciousness. Available online at: http://stanford.library.usyd.edu.au/entries/self-consciousness-phenomenological/

[B80] Gehrke B. J. Chefer V. I. Shippenberg T. S. (2008). Effects of acute and repeated administration of salvinorin A on dopamine function in the rat dorsal striatum. Psychopharmacology (Berl) 197, 509–517. 10.1007/s00213-007-1067-6 18246329 PMC3700373

[B81] Gennaro R. J. (2012). The Consciousness Paradox: Consciousness, Concepts, and Higher-Order Thoughts. Cambridge, MA: MIT Press.

[B82] Glennon R. A. Titeler M. McKenney J. D. (1984). Evidence for 5-HT2 involvement in the mechanism of action of hallucinogenic agents. Life Sci. 35, 2505–2511. 10.1016/0024-3205(84)90436-3 6513725

[B83] González D. Riba J. Bouso J. C. Gómez-Jarabo G. Barbanoj M. J. (2006). Pattern of use and subjective effects of salvia divinorum among recreational users. Drug Alcohol Depend. 85, 157–162. 10.1016/j.drugalcdep.2006.04.001 16720081

[B84] Gouzoulis-Mayfrank E. Habermeyer E. Hermle L. Steinmeyer A. Kunert H. Sass H. (1998). Hallucinogenic drug induced states resemble acute endogenous psychoses: results of an empirical study. Eur. Psychiatry 13, 399–406. 10.1016/S0924-9338(99)80686-5 19698655

[B85] Gouzoulis-Mayfrank E. Heekeren K. Neukirch A. Stoll M. Stock C. Obradovic M.. (2005). Psychological effects of (S)-ketamine and N,N-dimethyltryptamine (DMT): a double-blind, cross-over study in healthy volunteers. Pharmacopsychiatry 38, 301–311. 10.1055/s-2005-916185 16342002

[B86] Griffiths R. R. Johnson M. W. Richards W. A. Richards B. D. McCann U. Jesse R. (2011). Psilocybin occasioned mystical-type experiences: immediate and persisting dose-related effects. Psychopharmacology (Berl) 218, 649–665. 10.1007/s00213-011-2358-5 21674151 PMC3308357

[B87] Griffiths R. R. Richards W. A. McCann U. Jesse R. (2006). Psilocybin can occasion mystical-type experiences having substantial and sustained personal meaning and spiritual significance. Psychopharmacology (Berl) 187, 268–283; discussion 284–292. 10.1007/s00213-006-0457-5 16826400

[B88] Grof S. (1970). The use of LSD in psychotherapy. J. Psychedelic Drugs 3, 52–62. 10.1080/02791072.1970.10471362

[B89] Grosz E. (2015). “Being-in-the-world and schizophrenia,” in Psychology and the Other, eds Goodman D. Freeman M. (New York, NY: Oxford University Press), 247–269. Available online at: http://www.oxfordscholarship.com/view/10.1093/acprof:oso/9780199324804.001.0001/acprof-9780199324804-chapter-17

[B90] Gruhle H. W. (1915). Selbstschilderung und einfühlung. Z. Gesamte Neurol. Psychiatr. 27, 148–231. 10.1007/BF02866667

[B91] Guillot M. (2017). I me mine: on a confusion concerning the subjective character of experience. Rev. Philos. Psychol. 8, 23–53. 10.1007/s13164-016-0313-4 28386303 PMC5362670

[B92] Gusnard D. A. Akbudak E. Shulman G. L. Raichle M. E. (2001). Medial prefrontal cortex and self-referential mental activity: relation to a default mode of brain function. Proc. Natl. Acad. Sci. U S A 98, 4259–4264. 10.1073/pnas.071043098 11259662 PMC31213

[B93] Guterstam A. Björnsdotter M. Gentile G. Ehrsson H. H. (2015). Posterior cingulate cortex integrates the senses of self-location and body ownership. Curr. Biol. 25, 1416–1425. 10.1016/j.cub.2015.03.059 25936550

[B94] Guttmann E. Maclay W. S. (1937). Mescaline and depersonalization. J. Neurol. Psychopathol. 16, 193–212. 10.1136/jnnp.s1-16.63.193 PMC1038999 21610826

[B95] Halberstadt A. L. (2015). Recent advances in the neuropsychopharmacology of serotonergic hallucinogens. Behav. Brain Res. 277, 99–120. 10.1016/j.bbr.2014.07.016 25036425 PMC4642895

[B96] Halgren E. Walter R. D. Cherlow D. G. Crandall P. H. (1978). Mental phenomena evoked by electrical stimulation of the human hippocampal formation and amygdala. Brain 101, 83–115. 10.1093/brain/101.1.83 638728

[B97] Hasler F. Grimberg U. Benz M. A. Huber T. Vollenweider F. X. (2004). Acute psychological and physiological effects of psilocybin in healthy humans: a double-blind, placebo-controlled dose-effect study. Psychopharmacology (Berl) 172, 145–156. 10.1007/s00213-003-1640-6 14615876

[B98] Heydrich L. Blanke O. (2013). Distinct illusory own-body perceptions caused by damage to posterior insula and extrastriate cortex. Brain 136, 790–803. 10.1093/brain/aws364 23423672

[B99] Hohwy J. Michael J. (forthcoming). “Why should any body have a self?,” in The Subject’s Matter: Self-Consciousness and the Body, eds Alsmith A. Vignemont F. (Cambridge, MA: MIT Press).

[B100] Hood R. W. (2002). The mystical self: lost and found. Int. J. Psychol. Relig. 12, 1–14. 10.1207/s15327582ijpr1201_01

[B101] Huang H. C. Lee Y. T. Chen W. Y. Liang C. (2017). The sense of 1PP-location contributes to shaping the perceived self-location together with the sense of body-location. Front. Psychol. 8:370. 10.3389/fpsyg.2017.00370 28352241 PMC5348511

[B102] Husserl E. (1959). Erste Philosophie, 1923/24: Zweiter Teil: Theorie der phänomenologische Reduktion, (Vol.3) ed. Boehm R., (The Hague: Martinus Nijhoff).

[B103] Huxley A. (1954). The Doors of Perception and Heaven and Hell. London: Harper Collins Publishers.

[B104] Ionta S. Heydrich L. Lenggenhager B. Mouthon M. Fornari E. Chapuis D.. (2011). Multisensory mechanisms in temporo-parietal cortex support self-location and first-person perspective. Neuron 70, 363–374. 10.1016/j.neuron.2011.03.009 21521620

[B105] Javitt D. C. Steinschneider M. Schroeder C. E. Arezzo J. C. (1996). Role of cortical N-methyl-D-aspartate receptors in auditory sensory memory and mismatch negativity generation: implications for schizophrenia. Proc. Natl. Acad. Sci. U S A 93, 11962–11967. 10.1073/pnas.93.21.11962 8876245 PMC38166

[B106] Javitt D. C. Zukin S. R. Heresco-Levy U. Umbricht D. (2012). Has an angel shown the way? Etiological and therapeutic implications of the PCP/NMDA model of schizophrenia. Schizophr. Bull. 38, 958–966. 10.1093/schbul/sbs069 22987851 PMC3446214

[B107] Johnson M. W. MacLean K. A. Reissig C. J. Prisinzano T. E. Griffiths R. R. (2011). Human psychopharmacology and dose-effects of salvinorin A, a kappa opioid agonist hallucinogen present in the plant salvia divinorum. Drug Alcohol Depend. 115, 150–155. 10.1016/j.drugalcdep.2010.11.005 21131142 PMC3089685

[B108] Josipovic Z. Dinstein I. Weber J. Heeger D. J. (2012). Influence of meditation on anti-correlated networks in the brain. Front. Hum. Neurosci. 5:183. 10.3389/fnhum.2011.00183 22287947 PMC3250078

[B109] Kast E. C. Collins V. J. (1964). Study of lysergic acid diethylamide as an analgesic agent. Anesth. Analg. 43, 285–291. 10.1213/00000539-196405000-00013 14169837

[B110] Keefe R. S. Kraus M. S. Krishnan R. R. (2011). Failures in learning-dependent predictive perception as the key cognitive vulnerability to psychosis in schizophrenia. Neuropsychopharmacology 36, 367–368. 10.1038/npp.2010.153 21116261 PMC3055500

[B111] Klee G. D. (1963). Lysergic acid diethylamide (Lsd-25) and ego functions. Arch. Gen. Psychiatry 8, 461–474. 10.1001/archpsyc.1963.01720110037005 14033321

[B112] Kriegel U. (2005). Naturalizing subjective character. Phil. Phenomenol. Res. 71, 23–57. 10.1111/j.1933-1592.2005.tb00429.x

[B113] Kriegel U. (2009). Subjective Consciousness: A Self-Representational Theory. Oxford: Oxford University Press.

[B114] Krystal J. H. Karper L. P. Seibyl J. P. Freeman G. K. Delaney R. Bremner J. D.. (1994). Subanesthetic effects of the noncompetitive NMDA antagonist, ketamine, in humans: psychotomimetic, perceptual, cognitive, and neuroendocrine responses. Arch. Gen. Psychiatry 51, 199–214. 10.1001/archpsyc.1994.03950030035004 8122957

[B115] Lahti A. C. Koffel B. LaPorte D. Tamminga C. A. (1995). Subanesthetic doses of ketamine stimulate psychosis in schizophrenia. Neuropsychopharmacology 13, 9–19. 10.1038/sj.npp.1380271 8526975

[B116] Lane T. (2015). “Self, belonging, and conscious experience: a critique of subjectivity theories of consciousness,” in Disturbed Consciousness: New Essays on Psychopathology and Theories of Consciousness, ed. Rocco J. (Cambridge, MA: MIT Press), 103–139.

[B117] Leary T. Metzner R. Alpert R. (1964). The Psychedelic Experience: Manual Based on the Tibetan Book of the Dead. London: Penguin Classics.

[B118] Lebedev A. V. Kaelen M. Lövdén M. Nilsson J. Feilding A. Nutt D. J.. (2016). LSD-induced entropic brain activity predicts subsequent personality change. Hum. Brain Mapp. 37, 3203–3213. 10.1002/hbm.23234 27151536 PMC6867426

[B119] Lebedev A. V. Lövdén M. Rosenthal G. Feilding A. Nutt D. J. Carhart-Harris R. L. (2015). Finding the self by losing the self: neural correlates of ego-dissolution under psilocybin. Hum. Brain Mapp. 36, 3137–3153. 10.1002/hbm.22833 26010878 PMC6869189

[B120] Legrand D. (2006). The bodily self: the sensori-motor roots of pre-reflective self-consciousness. Phenomenol. Cogn. Sci. 5, 89–118. 10.1007/S11097-005-9015-6

[B121] Legrand D. Ruby P. (2009). What is self-specific? Theoretical investigation and critical review of neuroimaging results. Psychol. Rev. 116, 252–282. 10.1037/a0014172 19159156

[B122] Lemche E. Surguladze S. A. Brammer M. J. Phillips M. L. Sierra M. David A. S.. (2016). Dissociable brain correlates for depression, anxiety, dissociation, and somatization in depersonalization-derealization disorder. CNS Spectr. 21, 35–42. 10.1017/S1092852913000588 24059962

[B124] Lenggenhager B. Mouthon M. Blanke O. (2009). Spatial aspects of bodily self-consciousness. Conscious. Cogn. 18, 110–117. 10.1016/j.concog.2008.11.003 19109039

[B123] Lenggenhager B. Tadi T. Metzinger T. Blanke O. (2007). Video ergo sum: manipulating bodily self-consciousness. Science 317, 1096–1099. 10.1126/science.1143439 17717189

[B125] Letheby C. Gerrans P. (forthcoming). Self unbound: ego dissolution in psychedelic experience. Neurosci. Conscious. 10.1093/nc/nix016 PMC6007152 30042848

[B126] Levine J. (2001). Purple Haze: The Puzzle of Consciousness. (Vol. 6) Oxford: Oxford University Press.

[B127] Lewis D. J. Sloane R. B. (1958). Therapy with lysergic acid diethylamide. J. Clin. Exp. Psychopathol. 19, 19–31. 13525450

[B128] Limanowski J. (2014). What can body ownership illusions tell us about minimal phenomenal selfhood? Front. Hum. Neurosci. 8:946. 10.3389/fnhum.2014.00946 25505398 PMC4241829

[B129] Limanowski J. Blankenburg F. (2013). Minimal self-models and the free energy principle. Front. Hum. Neurosci. 7:547. 10.3389/fnhum.2013.00547 24062658 PMC3770917

[B130] Lou H. C. Changeux J. P. Rosenstand A. (2016). Towards a cognitive neuroscience of self-awareness. Neurosci. Biobehav. Rev. [Epub ahead of print]. 10.1016/j.neubiorev.2016.04.004 27079562

[B131] MacDonald A. A. Naci L. MacDonald P. A. Owen A. M. (2015). Anesthesia and neuroimaging: investigating the neural correlates of unconsciousness. Trends Cogn. Sci. 19, 100–107. 10.1016/j.tics.2014.12.005 25592916

[B132] Macpherson F. (2015). The structure of experience, the nature of the visual and type 2 blindsight. Conscious. Cogn. 32, 104–128. 10.1016/j.concog.2014.10.011 25481513

[B133] Maqueda A. E. Valle M. Addy P. H. Antonijoan R. M. Puntes M. Coimbra J.. (2015). Salvinorin-A induces intense dissociative effects, blocking external sensory perception and modulating interoception and sense of body ownership in humans. Int. J. Neuropsychopharmacol. 18:pyv065. 10.1093/ijnp/pyv065 26047623 PMC4675976

[B134] Maselli A. (2015). Allocentric and egocentric manipulations of the sense of self-location in full-body illusions and their relation with the sense of body ownership. Cogn. Process. 16, 309–312. 10.1007/s10339-015-0667-z 26220702

[B135] Mayer-Gross W. Stein H. (1926). Über einige abänderungen der sinnestätigkeit im Meskalinrausch. Z. Gesamte Neurol. Psychiatr. 101, 354–386. 10.1007/bf02878343

[B136] McGlothlin W. H. (1964). Hallucinogenic Drugs: A Perspective with Special Reference to Peyote and Cannabis. Ft. Belvoir: Defense Technical Information Center.

[B137] Metzinger T. (2003). Being No One: The Self-Model Theory of Subjectivity. Cambridge, MA: A Bradford Book.

[B138] Metzinger T. (2013). Why are dreams interesting for philosophers? The example of minimal phenomenal selfhood, plus an agenda for future research. Front. Psychol. 4:746. 10.3389/fpsyg.2013.00746 24198793 PMC3813926

[B139] Metzner R. Litwin G. Weil G. (1965). The relation of expectation and mood to psilocybin reactions: a questionnaire study. Psychedelic Rev. 5:339.

[B142] Mishara A. L. (2007). Is minimal self preserved in schizophrenia? a subcomponents view. Conscious. Cogn. 16, 715–721. 10.1016/j.concog.2007.07.009 17920523

[B140] Mishara A. Bonoldi I. Allen P. Rutigliano G. Perez J. Fusar-Poli P. (2016). Neurobiological models of self-disorders in early schizophrenia. Schizophr. Bull. 42, 874–880. 10.1093/schbul/sbv123 26385763 PMC4903042

[B141] Mishara A. L. Lysaker P. H. Schwartz M. A. (2014). Self-disturbances in schizophrenia: history, phenomenology, and relevant findings from research on metacognition. Schizophr. Bull. 40, 5–12. 10.1093/schbul/sbt169 24319117 PMC3885311

[B143] Moghaddam B. Adams B. Verma A. Daly D. (1997). Activation of glutamatergic neurotransmission by ketamine: a novel step in the pathway from NMDA receptor blockade to dopaminergic and cognitive disruptions associated with the prefrontal cortex. J. Neurosci. 17, 2921–2927. 9092613 10.1523/JNEUROSCI.17-08-02921.1997 PMC6573099

[B144] Mole C. (2008). Attention and consciousness. J. Conscious. Stud. 15, 86–104.

[B145] Morgan H. L. Turner D. C. Corlett P. R. Absalom A. R. Adapa R. Arana F. S.. (2011). Exploring the impact of ketamine on the experience of illusory body ownership. Biol. Psychiatry 69, 35–41. 10.1016/j.biopsych.2010.07.032 20947068 PMC3025328

[B146] Moutoussis M. Fearon P. El-Deredy W. Dolan R. J. Friston K. J. (2014). Bayesian inferences about the self (and Others): a review. Conscious. Cogn. 25, 67–76. 10.1016/j.concog.2014.01.009 24583455 PMC3989044

[B147] Musholt K. (2013). A philosophical perspective on the relation between cortical midline structures and the self. Front. Hum. Neurosci. 7:536. 10.3389/fnhum.2013.00536 24032013 PMC3759283

[B148] Muthukumaraswamy S. D. Carhart-Harris R. L. Moran R. J. Brookes M. J. Williams T. M. Errtizoe D.. (2013). Broadband cortical desynchronization underlies the human psychedelic state. J. Neurosci. 33, 15171–15183. 10.1523/JNEUROSCI.2063-13.2013 24048847 PMC6618409

[B149] Nagel T. (1974). What is it like to be a bat? Philos. Rev. 83, 435–450. 10.2307/2183914

[B151] Nelson B. Whitford T. J. Lavoie S. Sass L. A. (2014a). What are the neurocognitive correlates of basic self-disturbance in schizophrenia?: integrating phenomenology and neurocognition. Part 1 (Source monitoring deficits). Schizophr. Res. 152, 12–19. 10.1016/j.schres.2013.06.022 23810736

[B150] Nelson B. Whitford T. J. Lavoie S. Sass L. A. (2014b). What are the neurocognitive correlates of basic self-disturbance in schizophrenia?: integrating phenomenology and neurocognition. Part 2 (Aberrant salience). Schizophr. Res. 152, 20–27. 10.1016/j.schres.2013.06.033 23863772

[B152] Newell K. A. Zavitsanou K. Huang X. F. (2005). Ionotropic glutamate receptor binding in the posterior cingulate cortex in schizophrenia patients. Neuroreport 16, 1363–1367. 10.1097/01.wnr.0000174056.11403.71 16056140

[B153] Nichols D. E. (2004). Hallucinogens. Pharmacol. Ther. 101, 131–181. 10.1016/j.pharmthera.2003.11.002 14761703

[B154] Noel J. P. Cascio C. J. Wallace M. T. Park S. (2017). The spatial self in schizophrenia and autism spectrum disorder. Schizophr. Res. 179, 8–12. 10.1016/j.schres.2016.09.021 27650196 PMC5219859

[B155] Noel J. P. Pfeiffer C. Blanke O. Serino A. (2015). Peripersonal space as the space of the bodily self. Cognition 144, 49–57. 10.1016/j.cognition.2015.07.012 26231086 PMC4837893

[B157] Northoff G. Bermpohl F. (2004). Cortical midline structures and the self. Trends Cogn. Sci. 8, 102–107. 10.1016/j.tics.2004.01.004 15301749

[B156] Northoff G. Richter A. Bermpohl F. Grimm S. Martin E. Marcar V. L.. (2005). NMDA hypofunction in the posterior cingulate as a model for schizophrenia: an exploratory ketamine administration study in fMRI. Schizophr. Res. 72, 235–248. 10.1016/j.schres.2004.04.009 15560968

[B159] Nour M. M. Barrera A. (2015). Schizophrenia, subjectivity, and mindreading. Schizophr. Bull. 41, 1214–1219. 10.1093/schbul/sbv035 25848120 PMC4601706

[B160] Nour M. M. Carhart-Harris R. L. (2017). Psychedelics and the science of self-experience. Br. J. Psychiatry 210, 177–179. 10.1192/bjp.bp.116.194738 28249943

[B158] Nour M. M. Evans L. Nutt D. Carhart-Harris R. L. (2016). Ego-dissolution and psychedelics: validation of the ego-dissolution inventory (EDI). Front. Hum. Neurosci. 10:269. 10.3389/fnhum.2016.00269 27378878 PMC4906025

[B161] Nuller Y. L. Morozova M. G. Kushnir O. N. Hamper N. (2001). Effect of naloxone therapy on depersonalization: a pilot study. J. Psychopharmacol. 15, 93–95. 10.1177/026988110101500205 11448093

[B162] Ochsner K. N. Beer J. S. Robertson E. R. Cooper J. C. Gabrieli J. D. Kihsltrom J. F.. (2005). The neural correlates of direct and reflected self-knowledge. Neuroimage 28, 797–814. 10.1016/j.neuroimage.2005.06.069 16290016

[B163] Olney J. W. Labruyere J. Price M. T. (1989). Pathological changes induced in cerebrocortical neurons by phencyclidine and related drugs. Science 244, 1360–1362. 10.1126/science.2660263 2660263

[B164] Osmond H. (1957). A review of the clinical effects of psychotomimetic agents. Ann. N Y Acad. Sci. 66, 418–434. 10.1111/j.1749-6632.1957.tb40738.x 13425232

[B166] Pahnke W. N. (1969). The psychedelic mystical experience in the human encounter with death. Harv. Theol. Rev. 62, 1–21.

[B167] Pahnke W. N. (1966). Drugs and Mysticism: An Analysis of the Relationship between Psychedelic Drugs and the Mystical Consciousness. Cambridge, MA: Harvard University.

[B165] Pahnke W. N. Kurland A. A. Goodman L. E. Richards W. A. (1969). LSD-assisted psychotherapy with terminal cancer patients. Curr. Psychiatr. Ther. 9, 144–152. 5348915

[B168] Pahnke W. N. Richards W. A. (1966). Implications of LSD and experimental mysticism. J. Relig. Health 5, 175–208. 10.1007/BF01532646 24424798

[B169] Palhano-Fontes F. Andrade K. C. Tofoli L. F. Santos A. C. Crippa J. A. Hallak J. E. (2015). The psychedelic state induced by ayahuasca modulates the activity and connectivity of the default mode network. PLoS One 10:e0118143. 10.1371/journal.pone.0118143 25693169 PMC4334486

[B170] Pazos A. Probst A. Palacios J. M. (1987). Serotonin receptors in the human brain—IV. Autoradiographic mapping of serotonin-2 receptors. Neuroscience 21, 123–139. 10.1016/0306-4522(87)90326-5 3601071

[B171] Peckys D. Landwehrmeyer G. B. (1999). Expression of Mu, Kappa, and delta opioid receptor messenger RNA in the human CNS: a 33P in situ hybridization study. Neuroscience 88, 1093–1135. 10.1016/s0306-4522(98)00251-6 10336124

[B173] Petitmengin C. (2006). Describing one’s subjective experience in the second person: an interview method for the science of consciousness. Phenomenol. Cogn. Sci. 5, 229–269. 10.1007/s11097-006-9022-2

[B172] Petitmengin C. Lachaux J. P. (2013). Microcognitive science: bridging experiential and neuronal microdynamics. Front. Hum. Neurosci. 7:617. 10.3389/fnhum.2013.00617 24098279 PMC3784800

[B174] Petitmengin C. van Beek M. Bitbol M. Nissou J.-M. Roepstorff A. (forthcoming). What is it like to meditate? Methods and issues for a micro-phenomenological description of meditative experience. J. Conscious. Stud.

[B175] Pfeiffer A. Brantl V. Herz A. Emrich H. M. (1986). Psychotomimesis mediated by kappa opiate receptors. Science 233, 774–776. 10.1126/science.3016896 3016896

[B176] Pfeiffer C. Lopez C. Schmutz V. Duenas J. A. Martuzzi R. Blanke O. (2013). Multisensory origin of the subjective first-person perspective: visual, tactile, and vestibular mechanisms. PLoS One 8:e61751. 10.1371/journal.pone.0061751 23630611 PMC3632612

[B177] Prettyman A. (2014). Attention and conscious perception. Thesis. https://tspace.library.utoronto.ca/handle/1807/65560.

[B178] Qin P. Duncan N. Northoff G. (2013). Why and how is the self-related to the brain midline regions? Front. Hum. Neurosci. 7:909. 10.3389/fnhum.2013.00909 24399961 PMC3872301

[B179] Qin P. Northoff G. (2011). How is our self related to midline regions and the default-mode network? Neuroimage 57, 1221–1233. 10.1016/j.neuroimage.2011.05.028 21609772

[B180] Raichle M. E. MacLeod A. M. Snyder A. Z. Powers W. J. Gusnard D. A. Shulman G. L. (2001). A default mode of brain function. Proc. Natl. Acad. Sci. U S A 98, 676–682. 10.1073/pnas.98.2.676 11209064 PMC14647

[B181] Reissig C. J. Carter L. P. Johnson M. W. Mintzer M. Z. Klinedinst M. A. Griffiths R. R. (2012). High doses of dextromethorphan, an NMDA antagonist, produce effects similar to classic hallucinogens. Psychopharmacology (Berl) 223, 1–15. 10.1007/s00213-012-2680-6 22526529 PMC3652430

[B182] Riba J. Rodríguez-Fornells A. Barbanoj M. J. (2002). Effects of ayahuasca on sensory and sensorimotor gating in humans as measured by P50 suppression and prepulse inhibition of the startle reflex, respectively. Psychopharmacology (Berl) 165, 18–28. 10.1007/s00213-002-1237-5 12474114

[B183] Rosenthal D. M. (1997). “A theory of consciousness,” in The Nature of Consciousness, eds Block N. Flanagan O. J. Guzeldere G. (Cambridge, MA: MIT Press), 729–754.

[B184] Roth B. L. Baner K. Westkaemper R. Siebert D. Rice K. C. Steinberg S.. (2002). Salvinorin a: a potent naturally occurring nonnitrogenous kappa opioid selective agonist. Proc. Natl. Acad. Sci. U S A 99, 11934–11939. 10.1073/pnas.182234399 12192085 PMC129372

[B185] Rümmele W. Gnirss F. (1961). Untersuchungen mit psilocybin, einer psychotropen substanz aus psilocybe mexicana. Schweiz. Arch. Neurol. Neurochir. Psychiatr. 87, 365–385. 13744546

[B186] Sadzot B. Baraban J. M. Glennon R. A. Lyon R. A. Leonhardt S. Jan C. R.. (1989). Hallucinogenic drug interactions at human brain 5-HT2 receptors: implications for treating LSD-induced hallucinogenesis. Psychopharmacology (Berl) 98, 495–499. 10.1007/bf00441948 2505289

[B187] Salomon R. (2017). The assembly of the self from sensory and motor foundations. Soc. Cogn. 35, 87–106. 10.1521/soco.2017.35.2.87

[B188] Samad M. Chung A. J. Shams L. (2015). Perception of body ownership is driven by bayesian sensory inference. PLoS One 10:e0117178. 10.1371/journal.pone.0117178 25658822 PMC4320053

[B189] Sartre J.-P. (1943). L’être et Le Néant. Paris: Gallimard.

[B191] Sass L. Parnas J. Zahavi D. (2011). Phenomenological psychopathology and schizophrenia: contemporary approaches and misunderstandings. Philos. Psychiatr. Psychol. 18, 1–23. 10.1353/ppp.2011.0008

[B190] Sass L. Pienkos E. Nelson B. Medford N. (2013). Anomalous self-experience in depersonalization and schizophrenia: a comparative investigation. Conscious. Cogn. 22, 430–441. 10.1016/j.concog.2013.01.009 23454432

[B192] Savage C. (1955). Variations in ego feeling induced by D-lysergic acid diethylamide (LSD-25). Psychoanal. Rev. 42, 1–16. 14371878

[B193] Scharfetter C. (1981). Ego-psychopathology: the concept and its empirical evaluation. Psychol. Med. 11, 273–280. 10.1017/s0033291700052090 7267869

[B194] Scheidegger M. Walter M. Lehmann M. Metzger C. Grimm S. Boeker H.. (2012). Ketamine decreases resting state functional network connectivity in healthy subjects: implications for antidepressant drug action. PLoS One 7:e44799. 10.1371/journal.pone.0044799 23049758 PMC3461985

[B195] Schmidt A. Bachmann R. Kometer M. Csomor P. A. Stephan K. E. Seifritz E.. (2012). Mismatch negativity encoding of prediction errors predicts S-ketamine-induced cognitive impairments. Neuropsychopharmacology 37, 865–875. 10.1038/npp.2011.261 22030715 PMC3280661

[B196] Schneider K. (1959). Clinical Psychopathology. 5th Edn. New York, NY: Grune and Stratton.

[B197] Schwitzgebel E. (2007). Do you have constant tactile experience of your feet in your shoes? Or is experience limited to what’s in attention? J. Conscious. Stud. 14, 5–35.

[B198] Sedman G. Kenna J. C. (1964). The occurrence of depersonalization phenomena under LSD. Psychiatr. Neurol. (Basel) 147, 129–137. 10.1159/000128893 14134229

[B199] Serino A. Alsmith A. Costantini M. Mandrigin A. Tajadura-Jimenez A. Lopez C. (2013). Bodily ownership and self-location: components of bodily self-consciousness. Conscious. Cogn. 22, 1239–1252. 10.1016/j.concog.2013.08.013 24025475

[B201] Seth A. (2013). Interoceptive inference, emotion, and the embodied self. Trends Cogn. Sci. 17, 565–573. 10.1016/j.tics.2013.09.007 24126130

[B200] Seth A. K. Suzuki K. Critchley H. D. (2012). An interoceptive predictive coding model of conscious presence. Front. Psychol. 2:395. 10.3389/fpsyg.2011.00395 22291673 PMC3254200

[B202] Sharp F. R. Jasper P. Hall J. Noble L. Sagar S. M. (1991). MK-801 and ketamine induce heat shock protein HSP72 in injured neurons in posterior cingulate and retrosplenial cortex. Ann. Neurol. 30, 801–809. 10.1002/ana.410300609 1838680

[B205] Sierra M. (2008). Depersonalization disorder: pharmacological approaches. Expert Rev. Neurother. 8, 19–26. 10.1586/14737175.8.1.19 18088198

[B203] Sierra M. Baker D. Medford N. Lawrence E. Patel M. Phillips M. L.. (2006). Lamotrigine as an add-on treatment for depersonalization disorder: a retrospective study of 32 cases. Clin. Neuropharmacol. 29, 253–258. 10.1097/01.WNF.0000228368.17970.da 16960469

[B204] Sierra M. Phillips M. L. Lambert M. V. Senior C. David A. S. Krystal J. H. (2001). Lamotrigine in the treatment of depersonalization disorder. J. Clin. Psychiatry 62, 826–827. 10.4088/jcp.v62n1012b 11816874

[B206] Simeon D. Knutelska M. (2005). An open trial of naltrexone in the treatment of depersonalization disorder. J. Clin. Psychopharmacol. 25, 267–270. 10.1097/01.jcp.0000162803.61700.4f 15876908

[B207] Spence C. Driver J. (Eds). (2004). Crossmodal Space and Crossmodal Attention. 1st Edn. Oxford; New York: Oxford University Press.

[B208] Sterzer P. Mishara A. L. Voss M. Heinz A. (2016). Thought insertion as a self-disturbance: an integration of predictive coding and phenomenological approaches. Front. Hum. Neurosci. 10:502. 10.3389/fnhum.2016.00502 27785123 PMC5060939

[B209] Stiefel K. M. Merrifield A. Holcombe A. O. (2014). The claustrum’s proposed role in consciousness is supported by the effect and target localization of salvia divinorum. Front. Integr. Neurosci. 8:20. 10.3389/fnint.2014.00020 24624064 PMC3935397

[B211] Strawson G. (2000). “The phenomenology and ontology of the self,” in Exploring the Self, ed. Zahavi D. (Alphen aan den Rijn: Kluwer Academic Publishers), 23–39.

[B212] Strawson G. (2011). Selves: An Essay in Revisionary Metaphysics. Oxford: Oxford University Press.

[B210] Strassman R. J. Qualls C. R. Uhlenhuth E. H. Kellner R. (1994). Dose-response study of N,N-dimethyltryptamine in humans: II. Subjective effects and preliminary results of a new rating scale. Arch. Gen. Psychiatry 51, 98–108. 10.1001/archpsyc.1994.03950020022002 8297217

[B213] Studerus E. Gamma A. Kometer M. Vollenweider F. X. (2012). Prediction of psilocybin response in healthy volunteers. PLoS One 7:e30800. 10.1371/journal.pone.0030800 22363492 PMC3281871

[B214] Studerus E. Gamma A. Vollenweider F. X. (2010). Psychometric evaluation of the altered states of consciousness rating scale (OAV). PLoS One 5:e12412. 10.1371/journal.pone.0012412 20824211 PMC2930851

[B215] Studerus E. Kometer M. Hasler F. Vollenweider F. X. (2011). Acute, subacute and long-term subjective effects of psilocybin in healthy humans: a pooled analysis of experimental studies. J. Psychopharmacol. 25, 1434–1452. 10.1177/0269881110382466 20855349

[B216] Suzuki K. Garfinkel S. N. Critchley H. D. Seth A. K. (2013). Multisensory integration across exteroceptive and interoceptive domains modulates self-experience in the rubber-hand illusion. Neuropsychologia 51, 2909–2917. 10.1016/j.neuropsychologia.2013.08.014 23993906

[B217] Tagliazucchi E. Roseman L. Kaelen M. Orban C. Muthukumaraswamy S. D. Murphy K.. (2016). Increased global functional connectivity correlates with LSD-induced ego dissolution. Curr. Biol. 26, 1043–1050. 10.1016/j.cub.2016.02.010 27085214

[B218] Thakkar K. N. Nichols H. S. McIntosh L. G. Park S. (2011). Disturbances in body ownership in schizophrenia: evidence from the rubber hand illusion and case study of a spontaneous out-of-body experience. PLoS One 6:e27089. 10.1371/journal.pone.0027089 22073126 PMC3205058

[B219] Titeler M. Lyon R. A. Glennon R. A. (1988). Radioligand Binding Evidence Implicates the Brain 5-HT2 receptor as a site of action for LSD and phenylisopropylamine hallucinogens. Psychopharmacology (Berl) 94, 213–216. 10.1007/bf00176847 3127847

[B220] Uddin L. Q. (2011). Brain connectivity and the self: the case of cerebral disconnection. Conscious. Cogn. 20, 94–98. 10.1016/j.concog.2010.09.009 20875750 PMC3021584

[B221] Valenzuela Moguillansky C. O’Regan J. K. Petitmengin C. (2013). Exploring the subjective experience of the “rubber hand” illusion. Front. Hum. Neurosci. 7:659. 10.3389/fnhum.2013.00659 24167480 PMC3805941

[B222] Varela F. J. (1996). Neurophenomenology: a methodological remedy for the hard problem. J. Conscious. Stud. 3, 330–349.

[B400] Vermersch Pierre (1994). L’entretien d’explicitation. Paris: ESF.

[B223] Vollenweider F. X. Geyer M. A. (2001). A systems model of altered consciousness: integrating natural and drug-induced psychoses. Brain Res. Bull. 56, 495–507. 10.1016/s0361-9230(01)00646-3 11750795

[B224] Vollenweider F. X. Kometer M. (2010). The neurobiology of psychedelic drugs: implications for the treatment of mood disorders. Nat. Rev. Neurosci. 11, 642–651. 10.1038/nrn2884 20717121

[B227] Vollenweider F. X. Leenders K. L. Øye I. Hell D. Angst J. (1997a). DIfferential psychopathology and patterns of cerebral glucose utilisation produced by (*S*)- and (*R*)-ketamine in healthy volunteers using positron emission tomography (PET). Eur. Neuropsychopharmacol. 7, 25–38. 10.1016/s0924-977x(96)00042-9 9088882

[B225] Vollenweider F. X. Leenders K. L. Scharfetter C. Antonini A. Maguire P. Missimer J.. (1997b). Metabolic hyperfrontality and psychopathology in the ketamine model of psychosis using positron emission tomography (PET) and [18F]fluorodeoxyglucose (FDG). Eur. Neuropsychopharmacol. 7, 9–24. 10.1016/s0924-977x(96)00039-9 9088881

[B226] Vollenweider F. X. Leenders K. L. Scharfetter C. Maguire P. Stadelmann O. Angst J. (1997c). Positron emission tomography and fluorodeoxyglucose studies of metabolic hyperfrontality and psychopathology in the psilocybin model of psychosis. Neuropsychopharmacology 16, 357–372. 10.1016/s0893-133x(96)00246-1 9109107

[B228] Vollenweider F. X. Vollenweider-Scherpenhuyzen M. F. Bäbler A. Vogel H. Hell D. (1998). Psilocybin induces schizophrenia-like psychosis in humans via a serotonin-2 agonist action. Neuroreport 9, 3897–3902. 10.1097/00001756-199812010-00024 9875725

[B229] Von Mering O. Morimoto K. Hyde R. W. Rinkel M. (1957). “Experimentally induced depersonalization,” in Experimental Psychopathology, eds Hoch P. H. Zubin J. (New York, NY: Grune and Stratton), 66–77.

[B230] Walsh S. L. Strain E. C. Abreu M. E. Bigelow G. E. (2001). Enadoline, a selective kappa opioid agonist: comparison with butorphanol and hydromorphone in humans. Psychopharmacology (Berl) 157, 151–162. 10.1007/s002130100788 11594439

[B231] Ward A. M. Schultz A. P. Huijbers W. Van Dijk K. R. A. Hedden T. Sperling R. A. (2014). The parahippocampal gyrus links the default-mode cortical network with the medial temporal lobe memory system. Hum. Brain Mapp. 35, 1061–1073. 10.1002/hbm.22234 23404748 PMC3773261

[B232] Weber E. T. Andrade R. (2010). Htr2a gene and 5-HT2A receptor expression in the cerebral cortex studied using genetically modified mice. Front. Neurosci. 4:36. 10.3389/fnins.2010.00036 20802802 PMC2928707

[B233] Willmore-Fordham C. B. Krall D. M. McCurdy C. R. Kinder D. H. (2007). The hallucinogen derived from salvia divinorum, salvinorin A, has κ-opioid agonist discriminative stimulus effects in rats. Neuropharmacology 53, 481–486. 10.1016/j.neuropharm.2007.06.008 17681558

[B234] Windt J. M. (2010). The immersive spatiotemporal hallucination model of dreaming. Phenomenol. Cogn. Sci. 9, 295–316. 10.1007/s11097-010-9163-1

[B235] Windt J. M. (2015). Dreaming: A Conceptual Framework for Philosophy of Mind and Empirical Research. 1st Edn. Cambridge, MA: MIT Press.

[B236] Wolbach A. B. Jr. Miner E. J. Isbell H. (1962). Comparison of psilocin with psilocybin, mescaline and LSD-25. Psychopharmacologia 3, 219–223. 10.1007/bf00412109 14007905

[B237] Yu H. Li Q. Wang D. Shi L. Lu G. Sun L.. (2012). Mapping the central effects of chronic ketamine administration in an adolescent primate model by functional magnetic resonance imaging (fMRI). Neurotoxicology 33, 70–77. 10.1016/j.neuro.2011.11.001 22178134

[B238] Zahavi D. (2004). Phenomenology and the project of naturalization. Phenomenol. Cogn. Sci. 3, 331–347. 10.1023/b:phen.0000048935.94012.4e

[B239] Zahavi D. (2005). Subjectivity and Selfhood: Investigating the First-Person Perspective. Cambridge, MA: Bradford Book/MIT Press.

[B240] Zahavi D. (2010). “Minimal self and narrative self. A distinction in need of refinement,” in The Embodied Self: Dimensions, Coherence, and Disorders, eds Fuchs T. Sattel H. Heningnsen P. (Stuttgart: Schattauer), 3–11.

[B241] Zahavi D. (2014). Self and Other: Exploring Subjectivity, Empathy, and Shame. Oxford: Oxford University Press.

[B242] Zahavi D. Kriegel U. (2016). “For-me-ness: what it is and what it is not,” in Philosophy of Mind and Phenomenology: Conceptual and Empirical Approaches, eds Dahlstrom D. O. Elpidorou A. Hopp W. (London: Routledge), 36–53.

